# Arabidopsis Actin-Depolymerizing Factor-4 Links Pathogen Perception, Defense Activation and Transcription to Cytoskeletal Dynamics

**DOI:** 10.1371/journal.ppat.1003006

**Published:** 2012-11-08

**Authors:** Katie Porter, Masaki Shimono, Miaoying Tian, Brad Day

**Affiliations:** 1 Graduate Program in Cell and Molecular Biology, Michigan State University, East Lansing, Michigan, United States of America; 2 Department of Plant, Soil and Microbial Sciences, Michigan State University, East Lansing, Michigan, United States of America; National Institute of Biological Sciences, China

## Abstract

The primary role of Actin-Depolymerizing Factors (ADFs) is to sever filamentous actin, generating pointed ends, which in turn are incorporated into newly formed filaments, thus supporting stochastic actin dynamics. Arabidopsis ADF4 was recently shown to be required for the activation of resistance in Arabidopsis following infection with the phytopathogenic bacterium *Pseudomonas syringae* pv. tomato DC3000 (*Pst*) expressing the effector protein AvrPphB. Herein, we demonstrate that the expression of *RPS5*, the cognate resistance protein of AvrPphB, was dramatically reduced in the *adf4* mutant, suggesting a link between actin cytoskeletal dynamics and the transcriptional regulation of R-protein activation. By examining the PTI (PAMP Triggered Immunity) response in the *adf4* mutant when challenged with *Pst* expressing AvrPphB, we observed a significant reduction in the expression of the PTI-specific target gene *FRK1* (Flg22-Induced Receptor Kinase 1). These data are in agreement with recent observations demonstrating a requirement for RPS5 in PTI-signaling in the presence of AvrPphB. Furthermore, MAPK (Mitogen-Activated Protein Kinase)-signaling was significantly reduced in the *adf4* mutant, while no such reduction was observed in the *rps5-1* point mutation under similar conditions. Isoelectric focusing confirmed phosphorylation of ADF4 at serine-6, and additional *in planta* analyses of ADF4's role in immune signaling demonstrates that nuclear localization is phosphorylation independent, while localization to the actin cytoskeleton is linked to ADF4 phosphorylation. Taken together, these data suggest a novel role for ADF4 in controlling gene-for-gene resistance activation, as well as MAPK-signaling, *via* the coordinated regulation of actin cytoskeletal dynamics and *R*-gene transcription.

## Author Summary

The activation and regulation of the plant immune system requires the coordinated function of numerous pre-formed and inducible cellular responses. Following pathogen perception, plants not only activate specific defense-associated signaling, such as resistance (*R*) genes, but also redirect basic cellular machinery to support innate immune signaling. Within each of these processes, the actin cytoskeleton has been demonstrated to play a significant role in structural-based defense signaling in plants in response to pathogen infection. Most notably, the actin cytoskeleton of plants has been shown to play a role in structural-based defense signaling following fungal pathogen infection. Recent work from our laboratory has demonstrated that the actin cytoskeleton of Arabidopsis mediates defense signaling following perception of the phytopathogenic bacterium *Pseudomonas syringae*. Using a combination of genetic and cell biology-based approaches, we found that ADF4, a regulator of actin cytoskeletal dynamics, is required for the specific activation of *R*-gene-mediated signaling. By analyzing the activation of signaling following pathogen perception, we have identified substantial crosstalk between recognition of pathogen virulence factors (e.g., effector proteins) and the regulation of *R*-gene transcription. In total, our work highlights the intimate relationship between basic cellular processes and the perception and activation of defense signaling following pathogen infection.

## Introduction

The actin cytoskeleton is an essential, dynamic component of eukaryotic cells, involved in numerous processes including growth and development, cellular organization and organelle movement, and abiotic and biotic stress signaling [1]. Underpinning these processes in plants is a tightly regulated genetic and biochemical mechanism driven by the function of more than 70 actin-binding proteins (ABPs), which through their coordinated activity, regulates the balance of free globular (G)-actin versus filamentous (F)-actin, of which nearly 95% is unpolymerized in plants [2], [3]. As a consequence of this large pool of free G-actin, the potential exists for explosive rates of polymerization following elicitation by a broad range of external stimuli, including pathogen infection [1]. Among the numerous ABPs in plants responsible for modulating the balance of G- to F-actin, one subclass, Actin-Depolymerizing Factors (ADFs), both sever and disassemble F-actin. In addition to its primary role in modulating host cytoskeletal architecture, a role for ADFs in defense signaling following pathogen infection is emerging [4], [5], [6].

The initiation of innate immune signaling in plants relies on multiple pre-formed and inducible processes to surveil, respond, and activate defense signaling following pathogen perception [7], [8]. In total, these responses can be cataloged based on two primary nodes of defense signaling: pathogen-associated molecular pattern (PAMP)-triggered immunity (PTI) and effector-triggered immunity (ETI) [7]. In the case of PTI, perception and activation is typically mediated by extracellular plasma membrane-localized pattern recognition receptors (PRRs), which are responsible for the recognition of conserved pathogen motifs (i.e., PAMPs; e.g., flagellin, LPS, chitin). Recognition of PAMPs by PRRs initiates downstream signaling, including the activation of the Mitogen-Activated Protein Kinase (MAPK) signaling cascade, the generation of reactive oxygen species, and transcription of pathogen-responsive genes [9]. Arguably the best-characterized example of PTI signaling in plants is the activation of signaling associated with FLS2 (Flagellin Sensitive-2), a receptor-like kinase containing a serine/threonine kinase, which recognizes flagellin as well as the 22-amino acid peptide flg22 *via* the extracellular leucine rich repeat (LRR) domain [10], [11]. Activation of FLS2 by flg22 results in the association of FLS2 with BAK1 (BRI1-associated receptor kinase), as well as the phosphorylation of both FLS2 and BAK1 [12]. FLS2 ligand binding and association with BAK1 has been shown to activate the MAPK signaling pathway resulting in dual phosphorylation of conserved tyrosine and threonine residues of Arabidopsis (*Arabidopsis thaliana*) MAP kinases MPK3/6 [13], which in turn leads to transcription of PTI-related genes including *FRK1* (Flg22-induced receptor kinase 1; [14]). The expression of *FRK1*, however, is believed to be both MAPK dependent and independent [14].

As a counter to the activation of PTI, many plant pathogens deploy secreted effector proteins, which induce a host response (e.g., ETI) - an enhanced PTI-like response, as well as a more robust, programmed cell death-like, response known as the hypersensitive response (HR) that is initiated *via* the direct or indirect recognition of pathogen effectors by host resistance (R) proteins [7]. As expected, numerous virulence targets of pathogen effectors identified thus far are components of PTI signaling pathways – with the hypothesis being that targeting PTI-components can lead to increased virulence of the pathogen [9], [15]. Among the best-characterized signaling pathways leading to the activation of ETI, as well as a mechanistic example of the functional overlap between PTI and ETI, is the recognition of the bacterial effector protein AvrPphB by the Arabidopsis resistance protein RPS5 (resistance to *Pseudomonas syringae* 5) [7]. RPS5 is a member of the coiled-coil (CC) nucleotide-binding-site (NBS) LRR R-gene family, required for recognition of *Pseudomonas syringae* pv. tomato DC3000 (*Pst*) expressing the cysteine protease effector protein AvrPphB [16], [17]. RPS5-mediated resistance signaling is dependent upon AvrPphB cleavage of the receptor-like cytoplasmic kinase (RLCK) AvrPphB-Susceptible 1 (PBS1), which in turn results in the activation of ETI [18]. Recently, it has been suggested that the virulence target of AvrPphB may in fact be another RLCK, the PTI component BIK1 (*Botrytis*-induced kinase; [15]). This hypothesis is based on the observation that not only does AvrPphB cleave BIK1, as well as other RLCKs, including PBL1 (PBS1-like 1), but also that cleavage in the absence of RPS5 results in a significant reduction in PTI responses. It should be noted, that while the *bik1/pbl1* double mutant does have significant reductions in many PTI responses, *bik1/pbl1* does not exhibit reduced MPK3/6 phosphorylation upon flg22 stimulation [15], [19].

In the current study, we report the identification of a reduction in the expression and accumulation of *RPS5* mRNA in the absence of ADF4. In total, our data demonstrate that this reduction results in the down-regulation of PTI-signaling in the presence of the bacterial effector AvrPphB. Additionally, we demonstrate this reduction in PTI-signaling is due in part to an ADF4-dependent abrogation of the MPK3/6 branch of the MAPK pathway. From the standpoint of cellular dynamics and the activation of ETI, expression of *RPS5* was restored in an ADF4 phosphorylation-dependent manner, demonstrating a link between ADF4 phosphorylation, activity (e.g., F-actin binding), *RPS5* mRNA accumulation and subsequent resistance signaling. In addition to elucidating the signaling cascade from perception through MAPK activation, we identified a link between reduced actin cytoskeleton co-localization of ADF4 and the activation of RPS5-mediated resistance in a phosphorylation-dependent manner. In total, the work presented herein represents the first identification of link between the actin cytoskeleton, the dynamic control of ADF4, and the regulation of a resistance gene transcription.

## Results

### ADF4 is required for *RPS5* expression

**Figure 1:**
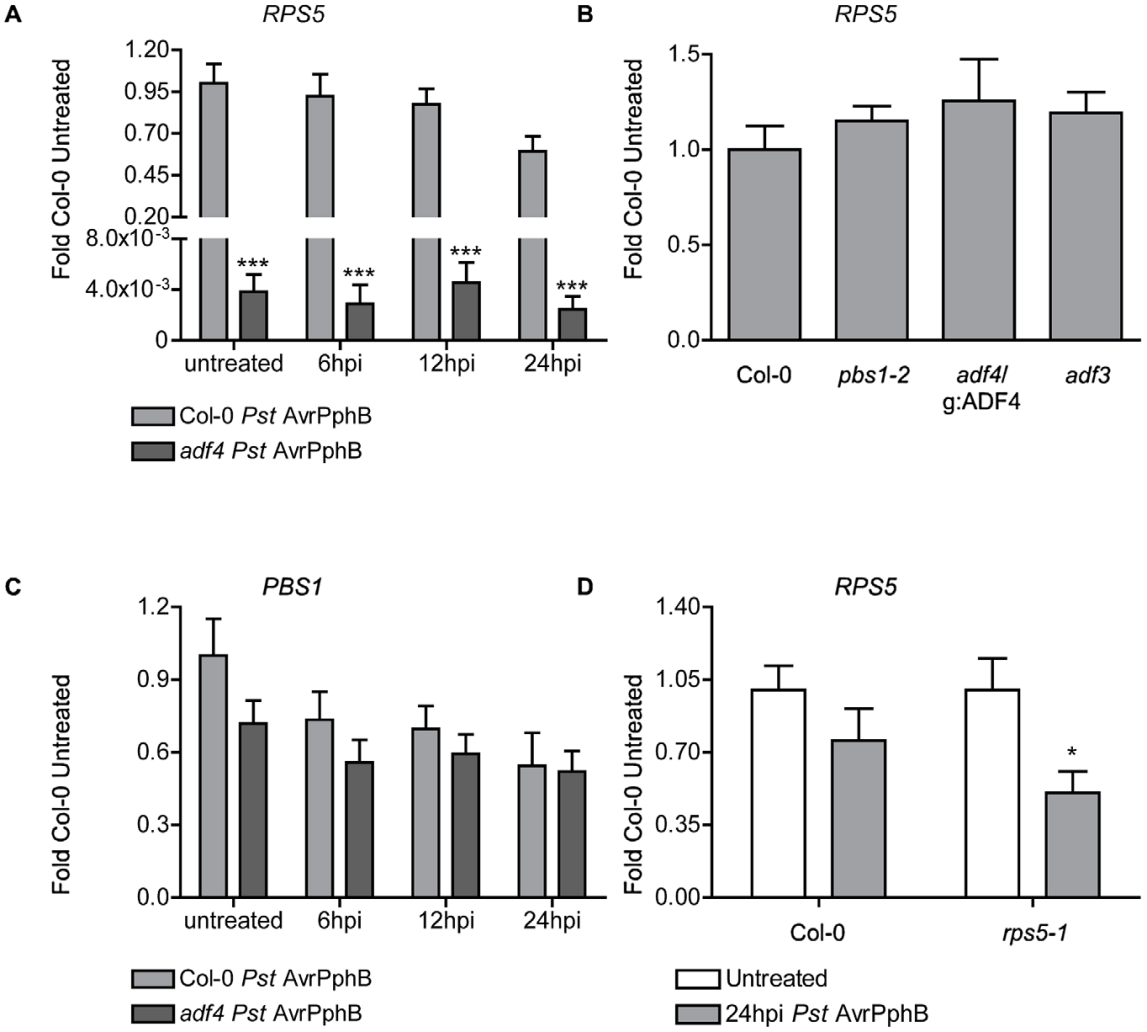
ADF4 is required for *RPS5* mRNA accumulation and resistance to *Pseudomonas syringae* expressing the cysteine protease effector AvrPphB. Time-course of mRNA accumulation of (A) *RPS5* and (C) *PBS1* in Col-0 and *adf4* mutant plants following dip inoculation with *Pst* AvrPphB. (B) Expression levels of *RPS5* in Col-0, *pbs1*, *adf4*/g:ADF4, and *adf3*. (D) *RPS5* mRNA accumulation in Col-0 and *rps5-1*, comparing each to their basal untreated levels at 24 hpi with *Pst* AvrPphB. Error bars represent mean ± SEM from two technical replicates of two independent biological repeats (n = 4). Statistical significance was determined using two-way ANOVA as compared to Col-0, with Bonferroni post test, where *p<0.05 and ***p<0.001. hpi = hours post inoculation.

Previous work has shown that Arabidopsis Actin-Depolymerizing Factor-4 (ADF4) is required for resistance to *Pst* AvrPphB, however, the biochemical and genetic mechanism(s) associated with activation were largely undefined [4]. To elucidate the signaling cascade leading from the recognition of AvrPphB to the activation of resistance, we first investigated the expression of the resistance (*R*) gene (i.e., *RPS5*) required for the recognition of AvrPphB. As shown in Figure 1A, we found a significant reduction (∼250-fold) in the accumulation of *RPS5* mRNA in the *adf4* mutant compared to wild-type Col-0. It was further determined that there is no significant alteration in the expression of *ADF4* in Col-0 during the course of infection with *Pst* AvrPphB (Figure S1). To address the possibility of positional effects in the *adf4* T-DNA SALK line, Tian et al. [4] demonstrated that complementation of the *adf4* mutant with native promoter-driven *ADF4* restored resistance to *Pst* AvrPphB. Similarly, these lines also showed a restoration in mRNA expression of *RPS5* (Figure 1B). The expression of *RPS5* in a second *ADF* mutant, *adf3*, was not altered (Figure 1B), confirming that the loss of resistance is specific to *ADF4*, as previously reported [4]. To confirm that the loss of RPS5-mediated resistance in the *adf4* mutant is specific to RPS5, we transformed the *adf4* mutant with a RPS5-sYFP (*adf4*/35S:RPS5-sYFP; [20]) to uncouple *RPS5* expression from native regulation. As shown in Figure S2, *RPS5* mRNA (Figure S2A) and HR-induced cell death following AvrPphB recognition (Figure S2B) was restored. Taken together, this data demonstrates a direct and specific requirement of ADF4 for RPS5-mediated resistance.

To determine the specificity of the ADF4-*RPS5* genetic interaction, we investigated if the mRNA expression of additional Arabidopsis *R*-genes are altered in the *adf4* mutant. To this end, we examined the expression of *RPS2* [21], *RPM1* [22], *RPS4* [23] and *RPS6* [24]. As an additional measure, we monitored the mRNA accumulation of *NDR1* (non race-specific disease resistance-1; [25], [26], [27]), a required component of most CC-NB-LRR defense signaling pathways in Arabidopsis, including RPS5. As shown in Figure S3, we did not observe a reduction in the resting levels of these mRNAs in the *adf4* mutant. To confirm that increased susceptibility and the loss of the HR in the *adf4* mutant is due to altered expression of *RPS5* (i.e., mRNA reduction) and not a reduction in the expression of the AvrPphB cleavage target PBS1 [16], [17], [28], [29], [30], the expression of *PBS1* mRNA was also measured. As shown in Figure 1C, we did not detect a significant difference between *PBS1* expression in the *adf4* mutant and Col-0. Additionally, there was no alteration of *RPS5* mRNA expression in the functional PBS1 mutant, *pbs1-2* ([30]; Figure 1B).

Our data present a role for ADF4 in the expression of *RPS5*, but not for the expression of *PBS1*, suggesting the loss of ETI in the *adf4* mutant may be a direct result of reduced *RPS5* expression (Figure 1A, Figure 1C). However, whether a role for AvrPphB in the down-regulation of *RPS5* expression exists is unknown. In order to address this question, we measured the expression of *RPS5* in both Col-0 and the RPS5 point-mutant, *rps5-1*; the rationale being that if AvrPphB negatively regulates the expression of *RPS5*, its expression should be reduced in the absence of the activation of ETI. In support of this hypothesis, as shown in Figure 1D, we observed a significant reduction in *RPS5* expression in *rps5-1* at 24 hpi following inoculation with *Pst AvrPphB*.

### The virulence activity of AvrPphB blocks MAPK signaling in *adf4*

**Figure 2:**
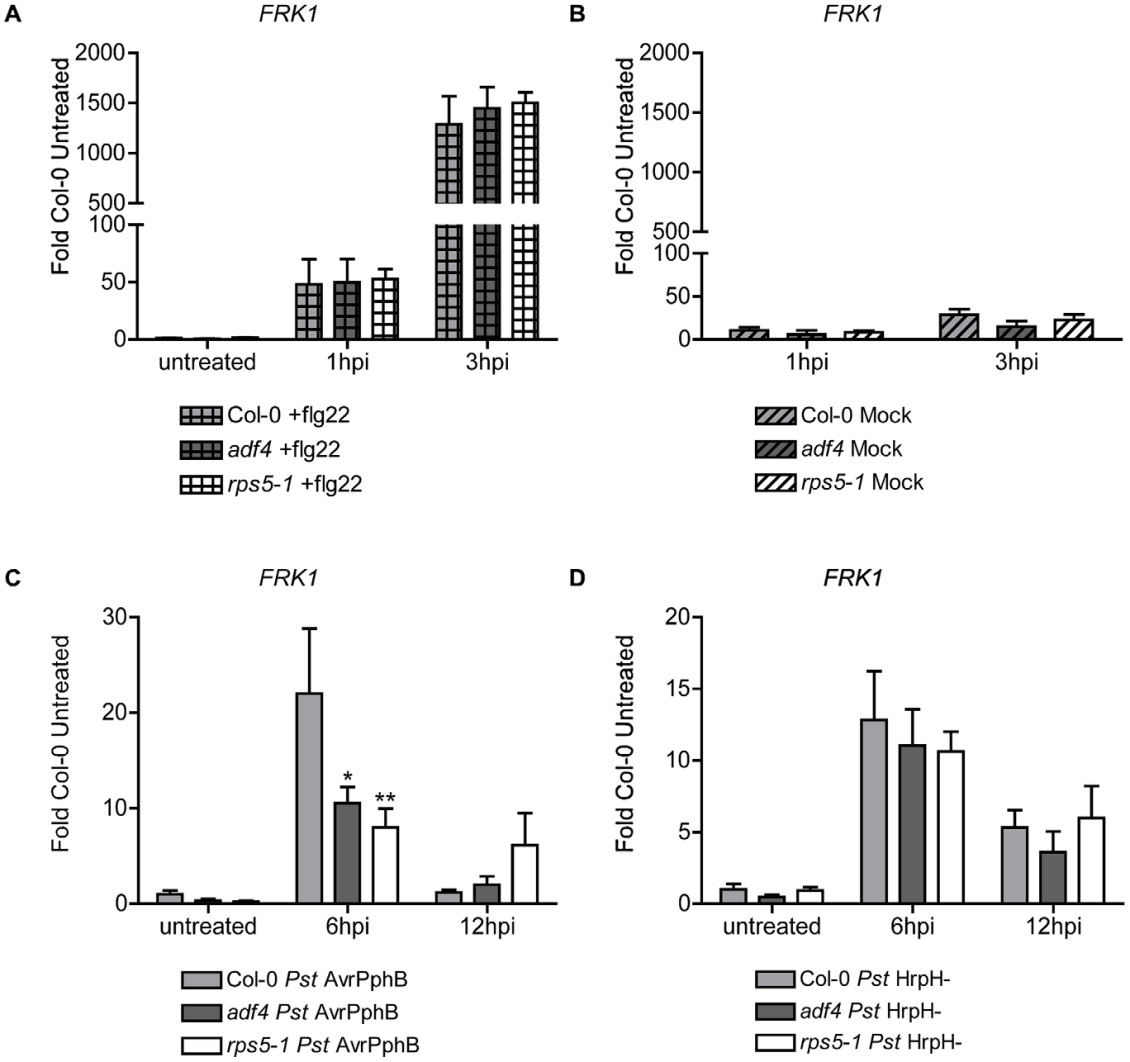
Flg22-induced receptor kinase 1 expression in the *adf4* mutant is reduced when the effector protein AvrPphB is expressed *in planta*. Relative expression levels of *FRK1* mRNA in Col-0, *adf4*, and *rps5-1* plants when treated with (A) 10 µM flg22, (B) mock inoculated with MgCl_2_ by hand infiltration (C) *Pst AvrPphB*, or (D) the *hrpH* ^−^ (*Pst hrpH* ^−^). Error bars represent mean ± SEM from two technical replicates of two independent biological repeats (n = 4). Statistical significance was determined using two-way ANOVA, as compared to Col-0, with Bonferroni post test where *p<0.05 and **p<0.005. hpi = hours post-inoculation.

Based on our observations above, we hypothesize that absence of RPS5-derived ETI in *adf4* is most likely due to the reduced expression of *RPS5*. Based on this, and given the significant overlap in signaling of ETI and PTI, particularly with regard to AvrPphB activity [9], [15], [31], we asked if PTI signaling is affected in the *adf4* mutant. To address this question, we first monitored the activation of *FRK1* expression, a transcriptional marker for FLS2 activation [14], in wild-type (WT) Col-0, *adf4* and *rps5-1*. As shown in Figure 2A, when Col-0, *adf4* and *rps5-1* plants were treated with flg22, no significant changes in *FRK1* mRNA expression were observed, and mock infiltration did little to activate *FRK1* (Figure 2A, Figure 2B). As a second, complementary analysis of the fidelity of PTI-based signaling responses in the *adf4* mutant, we also monitored root growth inhibition in the presence of flg22 (Chinchilla 2007, same as in the methods section). As shown in Figure S4, we did not observe a significant difference in root growth in *adf4* in the presence of flg22 as compared to Col-0. In total, these data demonstrate that flg22-induced PTI-signaling is functional in both the *rps5-1* and *adf4* mutants. As an additional measure to ensure that the technique employed in Figure 2A and B did not have an adverse effects on *RPS5* mRNA expression in either Col-0 or *adf4, RPS5* mRNA was monitored following hand-infiltration with either flg22 or mock (i.e., buffer alone). As shown in Figure S5A, we observed that flg22-induced expressional changes of *RPS5* mRNA was similar to that of mock, thus assuring the observed activation of *FRK1* in Col-0 and *adf4* (Figure 2A) can be attributed specifically to flg22, and is independent of the infiltration technique (Figure 2B), or changes in *RPS5* expression (Figure S5A).

Recent work from Zhang et al. [15] suggests that *FRK1* mRNA accumulation is reduced in the *rps5-1* mutant following flg22 treatment of protoplasts expressing AvrPphB. This raises the question of the relationship between the activation of PTI-signaling in parallel with the activation of ETI. To investigate the downstream signaling response(s) associated with the activation of RPS5-mediated resistance, we measured the expression of *FRK1* mRNA accumulation in Col-0, *adf4*, and *rps5-1* when inoculated with *Pst* AvrPphB. As shown in Figure 2C, we observed a significant decrease in *FRK1* mRNA expression in both the *adf4* and *rps5-1* mutants, as compared to Col-0, at 6 hpi with *Pst AvrPphB*. Coupled with the results of Zhang et al. [15], this would suggest that the *adf4* mutant has a decreased level of RPS5. In support of this, we did not detect a significant difference between *FRK1* expression in the *adf4* and *rps5-1* mutants when inoculated with flg22 (Figure 2A), demonstrating that the mutants had equivalent signaling potential following to FLS2 activation, and that ultimately, the reduction in *FRK1* expression is a direct result of a loss in ETI, most likely due to a reduction in *RPS5* mRNA expression and accumulation (Figure 1A).

It is possible that our observations described above could be an indirect result of cross-talk of PTI response signaling pathways in *adf4* and *rps5-1* in the presence of *Pst*. To test this, *FRK1* mRNA expression in Col-0, *adf4* and *rps5-1* following inoculation with the type three secretion system (T3SS) mutant *Pst hrpH* ^−^ was assessed to differentiate PTI from ETI in the *ADF4*-*RPS5* signaling node. As shown in Figure 2D, we detected no difference in *FRK1* mRNA expression between Col-0, *adf4* or *rps5-1*. Additionally, *RPS5* mRNA expression following *Pst hrpH* ^−^ inoculation (Figure S5B) and elf18-induced PTI-signaling in Col-0 and *adf4* (Figure S6) further supports these observations. When challenged with *Pst* expressing the catalytically inactive AvrPphB-C98S isoform [16], [18], both WT Col-0 and the *adf4* mutant showed increased expression levels of *FRK1* mRNA, in agreement with previously published data [15] (Figure S7A). A loss of induction of the HR in Col-0, *adf4* and *rps5-1* when challenged by *Pst* AvrPphB-C98S variant [18] confirms the catalytic inactivity of AvrPphB-C98S (Figure S7B).

**Figure 3:**
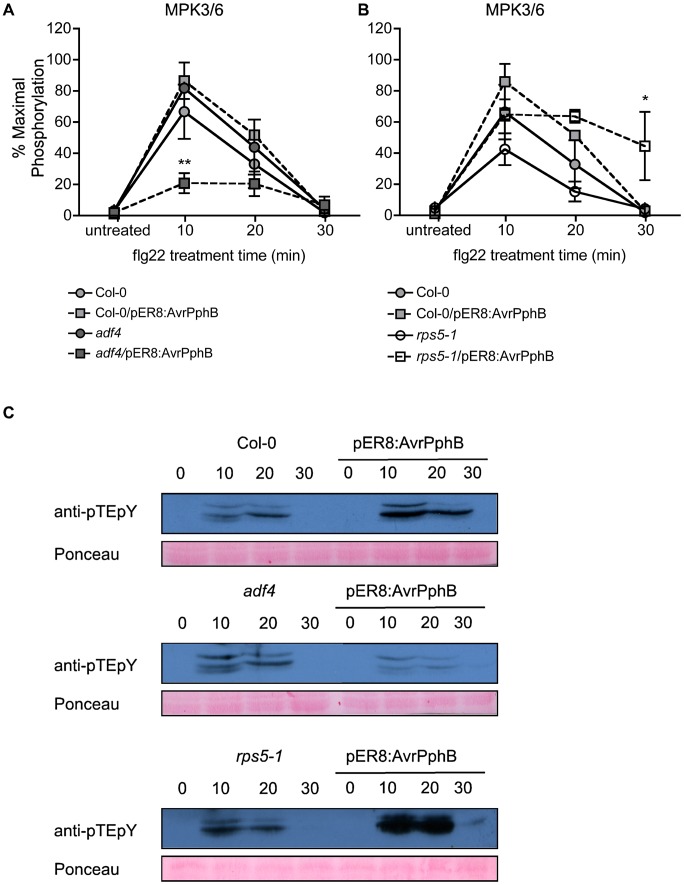
Mitogen Activated Protein Kinase (MAPK) phosphorylation is reduced in the *adf4* mutant in the presence of AvrPphB. (A) Percent maximal phosphorylation of the MPK3/6 TEY motif in Col-0 and the *adf4* mutant, +/− *AvrPphB*, followed by 1 µM flg22 treatment. (B) Percent maximal phosphorylation of the MPK3/6 TEY motif in Col-0 and the *rps5-1* mutant, +/− *AvrPphB*, followed by 1 µM flg22 treatment. *AvrPphB* expression was induced at 48 h pre-treatment with 100 µM estradiol in Col-0, *adf4* and *rps5-1* mutant plants containing an estradiol-inducible *AvrPphB* transgene (pER8:*AvrPphB*). Statistical significance was determined using two-way ANOVA as compared to Col-0 untreated, with Bonferroni post test, where *p<0.05, **p<0.005, n = 3. (C) Western blot analysis of MPK3/6 TEY phosphorylation.

At this point, we reasoned that altered *FRK1* expression in both the *rps5-1* and *adf4* mutants is due to a specific block in the MAPK signal cascade, most likely a function of the virulence activity of AvrPphB in the absence of ETI. To examine MAPK activation in the presence of both flg22 and AvrPphB, in the absence of pathogen, Col-0, *adf4* and *rps5-1* plants were transformed with an estradiol-inducible AvrPphB construct (i.e., Col-0/pER8:AvrPphB, *adf4*/pER8:AvrPphB and *rps5-1*/pER8:AvrPphB) to enable us to monitor the interplay between flg22 perception (i.e., PTI) and AvrPphB (i.e., ETI). As shown in Figure 3A and Figure 3C, when phosphorylation of both MPK3 and MPK6 was measured in response to flg22, a significant reduction in *adf4*/pER8:AvrPphB was observed as compared to Col-0 at 10 minutes; this reduction was not observed in *adf4*, and Col-0/pER8:AvrPphB. Interestingly, no significant reduction of MPK3 and MPK6 was observed in the *rps5-1*/pER8:AvrPphB 10 minutes after flg22 treatment (Figure 3B and Figure 3C). This observation suggests a potential combinatory role for ADF4 in both the expression of *RPS5* (Figure 1A), resulting in reduced PTI-signaling (Figure 2C), as well as in the proper regulation of MAPK-signaling in the presence of AvrPphB (Figure 3A and Figure 3C). Estradiol induction of *AvrPphB* is shown in Figure S8.

### Phosphorylated ADF4 is required for *RPS5* expression and subsequent activation of resistance

**Figure 4:**
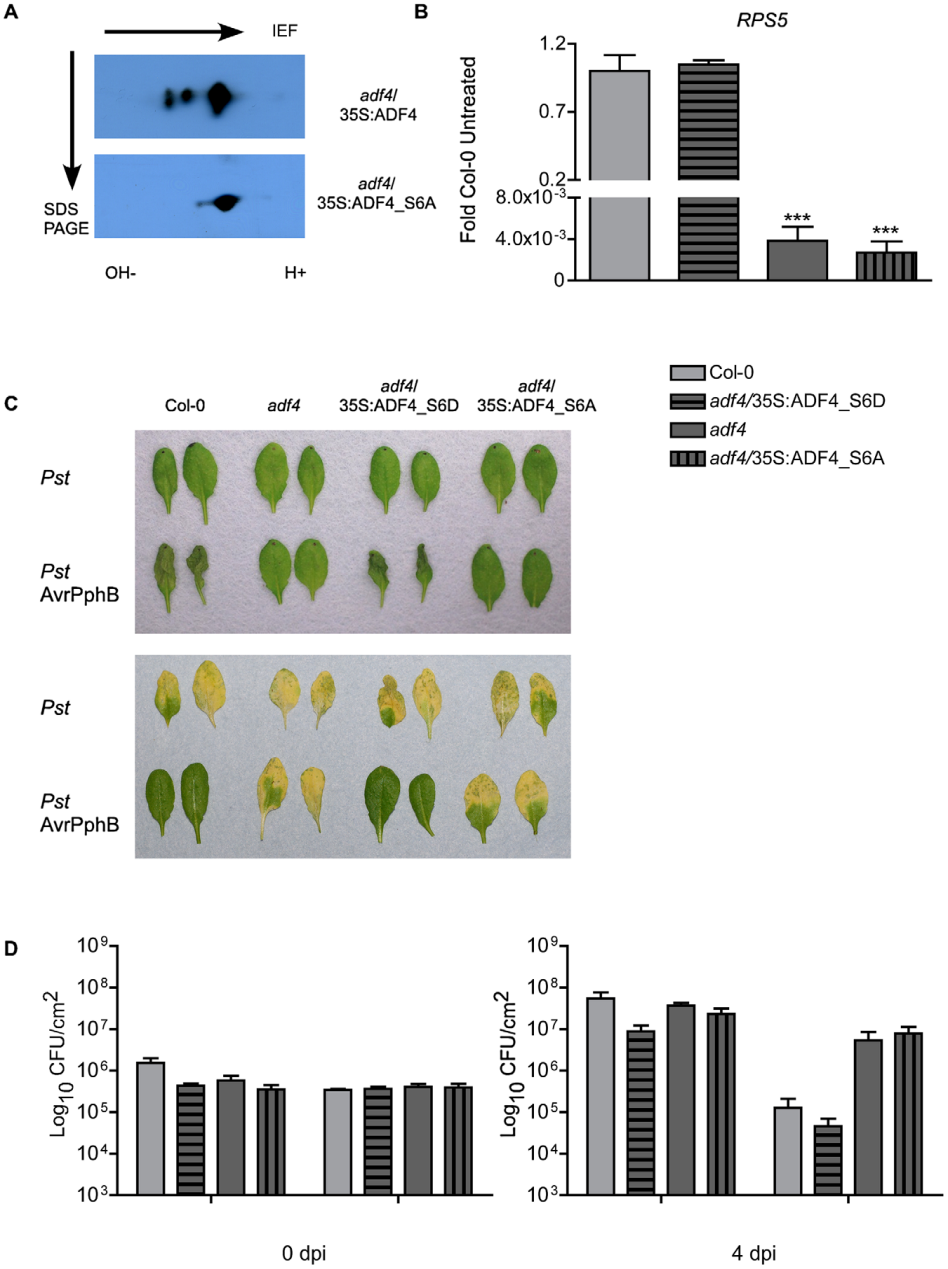
Phosphorylation of ADF4 is required for *RPS5* mRNA expression. (A) Western blot of isoelectric focusing (IEF) and SDS PAGE analysis of wild type ADF4 (upper) and phospho-null ADF4_S6A (lower). Arrows indicate direction of IEF and SDS PAGE. (B) The relative expression levels of *RPS5* were determined by qRT-PCR. (C) HR phenotypes at 22 hours after bacterial infiltration (upper), disease phenotypes at 4 dpi (lower). (D) Enumeration of bacterial growth at 0 and 4 dpi. HR and bacterial population experiments were repeated at least 3 times. Error bars, representing mean ± SEM, were calculated from two (A; n = 4) or three (D; n = 9) technical replicates of two independent biological repeats. Statistical significance was determined using two-way ANOVA, comparing *adf4* to Col-0, with Bonferroni post test, where *p<0.05; ***p<0.001. hpi = hours post inoculation; dpi = days post inoculation.

ADF4 mediated actin depolymerization is regulated in large part by the phosphorylation status of ADF. Indeed, previous work has demonstrated that mammalian cofilin/ADF activity is regulated by phosphorylation at serine-3, and that de/phosphorylation at this residue is responsible for the regulating the activation of actin depolymerization [32]. In plants, a direct correlation between the phosphorylation status of ADF and its function has not been demonstrated; however, ADF4 function is presumed to be regulated in a manner similar to that of mammalian cofilin [32], [33], [34]. Herein, we demonstrate for the first time that Arabidopsis ADF4 is indeed phosphorylated at serine-6, and that the phosphorylation status directly correlates with its activity and function of actin cytoskeletal dynamics. ADF4 and the phospho-null ADF4_S6A (i.e., serine-6 to alanine) plant lines were generated by expressing T7:ADF4 and T7:ADF4_S6A in the *adf4* mutant under the control of a constitutive promoter (*adf4*/35S:ADF4 and *adf4*/35S:ADF4_S6A). As shown in Figure 4A, after 2D isoelectric focusing (IEF) and SDS PAGE, native ADF4 shows a differential IEF profile than the phospho-null ADF4_S6A. In order to determine if phosphorylation of ADF4 affects *RPS5* expression, an additional phosphorylation isoform line was generated: a phospho-mimic isotype, reflecting a serine to aspartic acid change at amino acid position 6 (i.e., S6D) expressed in the *adf4* mutant background (*adf4*/35S:ADF4_S6D). As shown in Figure 4B, the phosphomimetic isoform, *adf4*/35S:ADF4_S6D, restored *RPS5* mRNA expression, while the phospho-null isoform, *adf4*/35S:ADF4_S6A, did not. A second independent transgenic Arabidopsis line expressing the ADF4 phosphorylation mutants were generated and tested for *RPS5* expression to ensure that altered mRNA expression was not due to a positional transgene insertion effect (Figure S9A).

To confirm that the ADF4 phosphomimetic constructs were functional in their ability to restore resistance in the *adf4* mutant, the induction of HR and disease phenotypes, as well as bacterial growth were assessed to determine the relationship between ADF4 phosphorylation and resistance activation through AvrPphB-RPS5. As shown in Figure 4C and 4D, inoculation of *adf4* mutant plants expressing the phosphomimetic (ADF4_S6D) with *Pst* AvrPphB restored the WT Col-0 resistance phenotype, both in terms of HR (Figure 4C, top panel), disease symptoms (Figure 4C, lower panel), and bacterial growth at 4 dpi (Figure 4D). Conversely, inoculation of the phospho-null-expressing plants (i.e., *adf4*/35S:ADF4_S6A) with *Pst* AvrPphB resulted in the absence of HR (Figure 4C, top panel), the development of disease symptoms (Figure 4C, lower panel), and an increase growth of the pathogen (Figure 4D), similar to that observed in the *adf4* mutant. As a control, to correlate transgene expression levels with our observations, the relative expression levels of both ADF4_S6A and ADF4_S6D were assessed by western blot to confirm that the observed restoration of *RPS5* with the phosphomimetic isoform was in fact due to the phosphorylation status and not an artifact of expression (Figure S9B). In total, our data confirms a restoration in resistance, as well as supports the hypothesis that phosphorylated ADF4 is required for resistance to *Pst* AvrPphB. Similarly, and in agreement our phosphorylation data, expression of *FRK1* following *Pst* AvrPphB inoculation in the *adf4*/35S:ADF4_S6D mutant was similar to that observed in Col-0, whereas the *adf4*/35S:ADF4_S6A plants had an *FRK1* expression pattern similar to the *adf4* mutant (Figure S10).

### Phosphorylation of ADF4 reduces its co-localization with F-actin, but does not influence nuclear targeting

**Figure 5:**
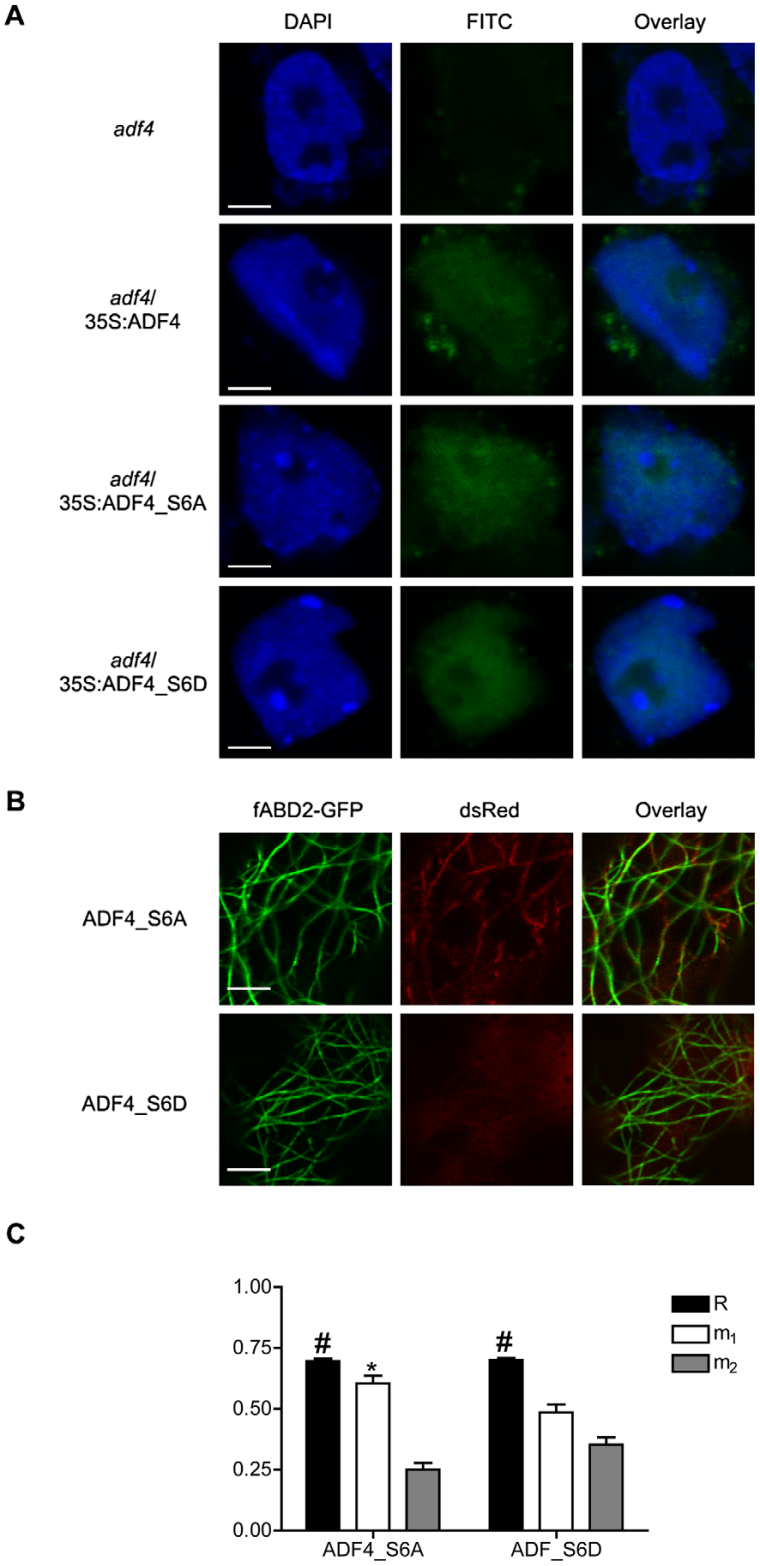
Confocal microscopy demonstrates phosphorylation of ADF4 affects cytoskeletal localization, but not nuclear localization. (A) Laser-scanning confocal microscopy of *adf4*, *adf4*/35S:ADF4, *adf4*/35S:ADF4_S6A and *adf4*/35S:ADF4_S6D isolated nuclei; DAPI stained nuclei (blue), immunochemistry FITC (green), and overlay. Bar = 2 µm. (B) Images of transiently expressed fABD2-GFP (green), dsRed- ADF4 _S6A/_S6D (red), and overlay in *Nicotiana benthamiana* taken by laser-scanning confocal microscopy. Bar = 5 µm. (C) Graphical representation of the overlay coefficient according to Manders (R) and the co-localization coefficients m_1_ and m_2_. Error bars, representing mean ± SEM, were calculated from two biological repeats (n = 40). Overlap coefficient (R) is considered to be co-localized when #R = 0.6 to 1.0, and co-localization coefficients indicate co-localization when *m_1_>0.5 and *m_2_>0.5.

As shown above, phosphorylated ADF4 is required for the accumulation of *RPS5* mRNA, as well as for resistance signaling in response to *Pst* AvrPphB (Figure 4). Previous work has demonstrated the potential for nuclear localization of ADFs, supportive of a role for actin and ADFs in regulating gene transcription [35], [36], [37]. To this end, we sought to determine if translocation of ADF4 into the nucleus is dependent upon the phosphorylation status of ADF4. As shown in Figure 5A, we found that ADF4, ADF4_S6A and ADF4_S6D are all present in the nucleus. This data would suggest that perturbation of *RPS5* expression in the *adf4*/35S:ADF4_S6A plants is not due to an inability of phospho-null ADF4 to enter the nucleus. However, the phospho-null ADF_S6A (ds-Red_ADF4) does show an increased co-localization with the actin cytoskeleton (filamentous Actin Binding Domain 2-GFP; fABD2-GFP), as well as the formation of filamentous like structures in the ADF4_S6A panel (Figure 5B). Conversely, phosphomimetic ADF4_S6D is more diffuse within the cytosol and has reduced co-localization with the actin cytoskeleton (Figure 5B).

To confirm our observations of a phosphorylation-specific alternation in the co-localization of our ADF4 isoforms (i.e., S6A *versus* S6D) with the actin cytoskeleton, we next performed a red-green analysis on the collected images, calculating the overlap coefficients, according to Manders (R). In short, this analysis will determine the actual overlap of the red/green signals in our collected images [38], providing an *in vivo* quantification of the co-localization of ADF4 with the actin cytoskeleton. As shown in Figure 5C, both ADF4_S6A and ADF4_S6D were found to have a significant R-value, 0.697±0.009 and 0.701±0.009 respectively, with significant differences in co-localization of ADF4_S6A and ADF4_S6D based on co-localization coefficients m_1_ and m_2_. For a red-green pairing, such as was preformed in our analysis, m_1_ refers to the fraction of red pixels co-localized with green pixels, while m_2_ is the fraction of green pixels co-localized with red pixels. The m_1_ values for ADF4_S6A and ADF4_S6D are 0.604±0.032 and 0.485±0.033 respectively, while the m_2_ values are 0.250±0.028 and 0.353±0.030 (Figure 5C). The co-localization coefficients suggest a significant co-localization of ADF_S6A with fABD2, but not for ADF4_S6D. In total, these observations are in agreement with previous reports of phosphorylated cofilin having reduced binding to both G- and F-actin [39].

## Discussion

**Figure 6:**
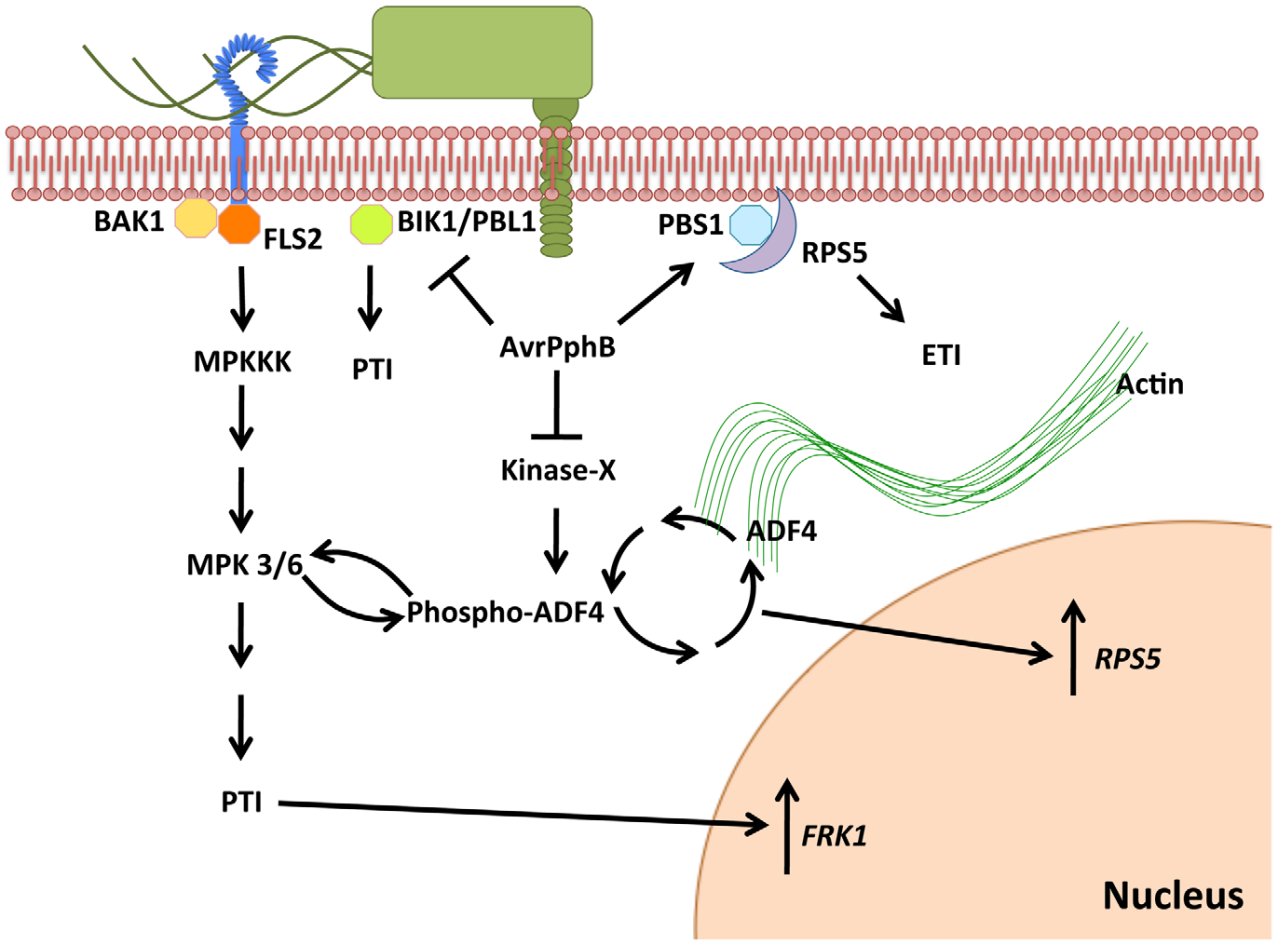
Proposed model illustrating the virulence and avirulence function of the bacterial cysteine protease AvrPphB through an ADF4-dependent mechanism. Following delivery of AvrPphB into the plant cells by *Pst* via the T3SS, AvrPphB targets multiple innate immune signaling pathways, including: 1) PTI, *via* the cleavage of BIK1 kinase; 2) ETI, *via* the cleavage of the kinase PBS1, a guardee of the resistance protein RPS5. We propose a potential role for AvrPphB in the modulation of actin cytoskeletal dynamics *via* the targeting of an unknown kinase responsible for the phosphorylation of ADF4 that ultimately results in reduced expression of *RPS5*, as well as specific down-regulation of MAP kinase signaling. ADF4 translocation into the nucleus is independent of phosphorylation status, however, F-actin co-localization and *RPS5* gene expression are dependent upon the phosphorylation of ADF4.

Understanding the mechanism(s) of pathogen effector recognition, as well as elucidating the putative virulence function(s) of these secreted proteins, provides the foundation for our understanding of innate immune signaling in plants [8]. Using a combination of cell biology, biochemical, and genetics-based approaches, we show that ADF4 is required for the specific activation of RPS5-mediated resistance. In both plants and animals, the actin cytoskeletal network plays a broad role in numerous cellular processes, including cell organization, growth, development and response to external stimuli, including pathogen infection. Herein, we propose a mechanism through which the expression of the *R*-gene *RPS5* is under the control of the actin binding protein ADF4, in a phosphorylation dependent manner, independent of nuclear localization, which subsequently affects co-localization with actin, suggesting a possible cytoskeletal role in gene transcription (Figure 6).

In animal cells, a complex signaling network involving Rho-GTPase activation, actin cytoskeletal dynamics, and the interplay between pathogen virulence has been extensively characterized [1]. In plants, however, the elucidation of the genetic link between pathogen virulence and the regulation of actin cytoskeletal dynamics has only recently been described [4], [5]. In plant-pathogen interactions, the effects of modulation to the host actin cytoskeleton have been best characterized using a combination of pharmacological and cell biology-based approaches to monitor focal orientation of F-actin filaments to the site of infection during fungal pathogenesis [6], [40], [41], [42], [43]. As a first step towards elucidating the mechanism of activation of RPS5-meditated resistance, we examined the expression levels of Arabidopsis genes associated with resistance to *Pst* AvrPphB. We observed a marked reduction in mRNA levels of the *R*-gene *RPS5*, while the protein kinase PBS1 was not affected (Figure 1B, Figure 1C). Additionally, the mRNA levels of *R*-genes unrelated to the recognition of AvrPphB were not affected in the *adf4* mutant (Figure S2B). From these data, we conclude that ADF4 is specifically required for the expression of *RPS5* and subsequent resistance to *Pst* AvrPphB.

The initiation of resistance signaling in plants following pathogen infection engages a multitude of processes, including PRR activation [12], MAPK signaling [14] and transcriptional reprogramming [44]. In the current study, our observation of a reduction in PTI-signaling in the *adf4* mutant supports our hypothesis that *RPS5* mRNA levels correlate with reduced levels of RPS5 protein. In support of this, we observed a reduction in *FRK1* transcript accumulation in the presence of AvrPphB in both the *adf4* and *rps5-1* mutants. This observation is in agreement with recent reports, including a study demonstrating a physical interaction between FLS2 and RPS5, which would suggest that PTI and ETI signaling is more interdependent than previously hypothesized [45]. Subsequent analysis of upstream MAPK components partially attributed diminished *FRK1* mRNA levels to a reduced activation of MPK3/6. Herein, we did not detect a significant reduction in flg22-induced phosphorylation of MPK3/6 in either Col-0/pER8:AvrPphB or *rps5-1*/pER8:AvrPphB; however, in *adf4*/pER8:AvrPphB plants, a significant reduction in MPK3/6 phosphorylation following flg22 treatment was observed (Figure 3). MAPK signaling is often primarily associated with PTI (i.e. flagellin activation of the FLS2 receptor); however, many reports have demonstrated the necessity of these components for ETI. For example, in tomato (*Solanum lycopersicum*) and tobacco (*Nicotiana tabacum*) the requirement of MAPK signaling-components for AvrPto- and N-mediated ETI has been well documented [46], [47], [48]. Our data would suggest that in the case of AvrPphB, R-Avr activation does not specifically induce MPK3/6 within 48 hours of estradiol-induced expression of AvrPphB (Figure 3B). Furthermore, the absence of perturbation to MPK3/6 in the *rps5-1*/pER8:AvrPphB suggest that while it appears recognition is important for aspects of PTI-signaling i.e. *FRK1* mRNA expression (Figure 2C), MAPK-signaling specifically is independent of the need for recognition (Figure 3B).

One possible explanation for reduced MAPK-signaling in the absence of ADF4 reflects the virulence activity of AvrPphB. Indeed, recent work has demonstrated a physical interaction between BIK1 and the FLS2 receptor upon ligand activation – an association that is required for the activation of PTI-signaling [15]. As a mechanism linking with the virulence activity of AvrPphB with both PTI and ETI, cleavage of BIK1 by AvrPphB results in reduced PTI-signaling in the absence of recognition (i.e. the *rps5-1* mutant). Our observation of a reduction in MPK3/6 phosphorylation in *adf4*, but not Col-0 nor *rps5-1*, would suggest an additional role for ADF4 in regulation of MAPK-signaling, while the reduced *FRK1* in *adf4* and *rps5-1* as compared to Col-0, supports the aforementioned potential virulence activity of AvrPphB, as well as a possible role for recognition (i.e. ETI) in the protection/recovery of the targeted PTI-signaling pathway. Although the mechanism(s) utilized by Arabidopsis to preserve the integrity of the MAPK- and PTI-signaling pathway are not yet fully understood, it is possible that ETI-induced SA accumulation, which has been demonstrated to prime and enhance accumulation of MPK3/6, can be partially responsible for the recovery of MAPK signaling in Col-0 [49]. Another possible contribution to the reduction in PTI-signaling associated with loss of ETI is the aforementioned direct association of FLS2 with RPS5 [45].

In plants, ADF localization is intimately associated with actin reorganization [50]. At present, a full understanding of how translocation of ADFs into the nucleus occurs has not been defined [51]; moreover, the precise function within the nucleus is unclear [36]. The current hypothesis is the translocation of ADFs, as well as other ABPs, into the nucleus may serve a chaperone function [39]. In support of this, actin, as well as several actin-binding proteins (including ADFs), has recently been shown to be present in the nuclei of Arabidopsis [36]. This data support the hypothesis that in addition to actin, ABPs and actin-related proteins (ARPs) may have specific functions within the nucleus, including chromatin assembly and remodeling, as well as participation in various steps of RNA transcription and processing [36], [52]. It is quite possible that ADF4 either facilitates nuclear translocation of specific actin isoforms required for processes related to the expression of *RPS5*, or, ADF4 itself is required for gene expression (i.e., transcription), as has been demonstrated to be the cased for other ARPs. Mechanistically, however, it is unclear how ADF proteins are translocated into the nucleus. Plant ADFs do not have a conserved nuclear localization signal sequence, as is found in the vertebrate ADFs/cofilins; however, plant ADFs do have two regions with basic amino acids which are similar to domains in other plant proteins that function together as a nuclear localization signal (NLS) [53]. To date, the function of these domains has not been explored. Our data, as well as a recent study by Kandasamy et al. [36], suggests that these two regions of basic amino acids may be both sufficient for translocation to the nucleus, which is not affected by the phosphorylation status of ADF4 at serine-6 (Figure 5).

In the current study, we demonstrate that ADF4 phosphorylation influences both actin cytoskeletal localization, and ultimately, *RPS5* mRNA expression (Figure 4, Figure 5). In total, our data provide *prima facie* evidence for an actin-based regulatory mechanism controlling *R*-gene expression, and further support the emerging hypothesis that there are critical physiological roles for phosphorylated ADFs in plants [39]. Phosphorylation of cofilin, the predominant ADF found in animal cells, is regulated in part through the action of LIM kinase [54], and results in a reduced affinity of cofilin for F-actin. To this end, ADF phosphorylation has commonly been viewed as an inactivation mechanism, however, recent data suggest that this is not the case [39]. In plant-pathogen interactions, numerous defense-associated processes are regulated by kinase phosphorylation [15], [18], [55], [56]. Conversely, the regulatory mechanisms controlling the phosphorylation, and subsequent regulation of actin dynamics, have not been well established, nor has the crosstalk between ADF regulation and innate immune signaling been fully defined. One obvious disconnect in the link between innate immune signaling and kinase activity in plants and animals is that plants do not have a kinase family homologous to mammalian LIM kinases [54], and thus, ADF phosphorylation is likely mediated by the activity of additional kinase(s), such as calcium dependent protein kinases [33]. One interesting hypothesis in support of the work described herein is that the kinase responsible for the phosphorylation of ADF4 may be a virulence target of AvrPphB. This hypothesis is supported in part by Figure 1D, in which *RPS5* expression is significantly reduced in the *rps5-1* point mutant following inoculation with *Pst* AvrPphB. Additionally, the observed requirement of ADF4 for MAPK-signaling in the presence of AvrPphB (Figure 3A) lends support for the idea of ADF4, or the kinases required for its regulation as potential virulence targets. In this regard, such a mechanism would further solidify a link between the virulence function and activity of AvrPphB and the role of the actin cytoskeleton in controlling *RPS5* transcription and disease signaling.

## Materials and Methods

### Plant growth, transformation, and bacterial growth assays

Arabidopsis plants were grown in a BioChambers walk-in growth chamber (model FLX-37; Winnipeg, Manitoba, Canada) at 20°C under a 12-hour light/12-hour dark cycle, with 60% relative humidity and a light intensity of 100 µmol photons m^−2^s^−1^. Transformation of Arabidopsis, as well as selection of transformants, was performed as described by Clough and Bent [57].

*Pseudomonas syringae* pv. tomato DC3000 (*Pst*) strains were grown as previously described [4]. Four-week-old plants were used for bacterial inoculations. For growth assays and qRT-PCR analyses, whole plants were dip inoculated into bacterial suspensions of 3×10^8^ colony-forming units (cfu) mL^−1^ in 10 mM MgCl_2_ containing 0.1% Silwet L-77. Three 0.7 cm diameter leaf disks were collected from three plants for bacterial growth assays, as previously described [4]. The hypersensitive response (HR) was analyzed by hand infiltrating bacterial suspension in 10 mM MgCl_2_ at 5×10^7^ cfu mL^−1^ and scoring leaves for tissue collapse 20 to 24 hours post inoculation.

flg22 infiltration was performed at a concentration of 1–10 µM in 10 mM MgCl_2_, as previously described [27]. Col-0 and *adf4* plants were grown upright on plates containing MS media for 10 days±10 nM flg22 in a GC8-2H growth chamber (Environmental Growth Chambers LTD., Winnipeg, Manitoba, Canada) at 20°C under a 12-hour light/12-hour dark cycle, with 60% relative humidity and a light intensity of 120 µmol photons m^−2^s^−1^. Analysis of flg22 inhibition of root growth was performed as previously described [12].

### Plasmid construction

The native promoter driven pMD1-g:*ADF4* (g:*ADF4*) was constructed as described in Tian et al. [4]. Primer sequences 5′-GCGGTCGACATGGCTAATGCTGCGTCAGGAATGG-3′ (forward ADF4), 5′-GCGGTCGACATGGCTAATGCTGCGGCAGGAATGG-3′ (forward ADF4_S6A), 5′-GCGGTCGACATGGCTAATGCTGCGGACGGAATGG-3′ (forward ADF4_S6D) and 5′- GCGGTCGACATGGCTAATGCTGCGTCAGGAATGG -3′ (reverse for all 3) were used to add *Sal*I restriction enzyme sites (underlined) for cloning *ADF4* and its phospho-mutants into pMD1:35S:T7 [27].

### Nuclei isolation and immunocytochemistry

Nuclei isolations were conducted as described in Kandasamy et al. [36]. Approximately 1 g of 2- to 3-week old *adf4*/35S:ADF4, _S6A, and _S6D Arabidopsis seedlings, grown upright on MS medium plates were used for each nuclear extraction. The isolated nuclei were fixed on chrome alum slides, permeabilized, and incubated with primary antibody T7-monoclonal (EMD Chemicals, Gibbstown, NJ, USA), secondary anti-mouse IgG-FITC (Sigma-Aldrich) and DAPI (Sigma-Aldrich) before imaging [36].

### Laser-scanning confocal microscopy and co-localization analysis

Isolated nuclei and transiently expressed dsRed-ADF4 constructs, and fABD2-GFP generated using *Agrobacterium tumefaciens*-mediated transient expression in *Nicotiana benthamiana*, were imaged using laser confocal scanning microscopy using a 60×/1.42 PlanApo N objective on an Olympus FV1000 (Olympus America Inc, Center Valley, PA), as described in Tian et al. [58]. Co-localization was preformed utilizing FluoView FV1000 (System Analysis Software, Olympus). An area of each image was selected for analysis containing <50% fABD2-GFP occupancy in order to examine true co-localization and not artificial co-localization due to over abundance of fABD2-GFP. Thresholds were set manually to account for background, and overlap coefficient according to Manders (R), and co-localization coefficients m1 and m2 were generated by the FV1000-ASW. Co-localization coefficient equations used can be found in Table S1.

### RNA isolation and qRT-PCR analysis

Total RNA was extracted from leaves using the PrepEase Plant RNA Spin kit (USB Affymetrix, Santa Clara, CA, USA). First-strand cDNA was synthesized from 1 µg total RNA using the First-Strand cDNA Synthesis kit (USB Affymetrix). Primers used for quantitative real-time PCR (qRT-PCR) are listed in Table S2. qRT-PCR was performed using the Mastercycler ep Realplex system (Eppendorf AG, Hamburg, Germany), as previously described [27], using the Hot Start SYBR Master mix 2× (USB Affymetrix). Ubiquitin (*UBQ10*) was used as an endogenous control for amplification. Fold Col-0 was determined using the following equation: (relative expression)/(relative expression of Col-0 untreated), where “relative expression” = 2^(−ΔCt)^, where ΔCt = *Ct_gene of interest_*−Ct*_UBQ10_*.

### Statistical analysis

All data were analyzed using GRAPHPAD PRISM Software (San Diego, California, USA). Values are represented as mean ±SEM. All statistical analysis was performed using two-way ANOVA, followed by the Bonferroni post-test as compared to Col-0. In Figure 2C, a two-way ANOVA, followed by the Bonferroni post-test was performed in order to determine if there is a significant difference between *rps5-1* and *adf4*. In Figure S1, an unpaired student t-test with a 95% confidence interval was performed to determine if change over time was significant. P values≤0.05 are considered significant, where *p<0.05; **p<0.01 and ***p<0.005.

### Immunoblot analysis

Western blot analysis of phosho-MPK3/6 was performed using 40 µg total protein, utilizing anti-pTEpY (Cell Signaling Technology, Danvers, MA, USA), while analysis of *adf4*/35S:ADF4_S6A and *adf4*/35S:ADF4_S6D was preformed using 20 µg total protein, utilizing anti-T7-HRP (EMD Chemicals, Gibbstown, NJ, USA), as previously described [26].

2D IEF was preformed on 500 mg of total lysate from *adf4*/35S:ADF4 and *adf4*/35S:ADF4_S6A. The lysates were precipitated using chloroform∶methanol (1∶4) and reconstituted in Urea buffer (7 M Urea, 2 M Thiourea, 2% CHAPS, 2% ASA-14, 50 mM DTT, 0.2% Biolyte ampholytes and 0.1% bromophenol blue). Isoelectric focusing was conducted according to manufacturing guidelines at the proteomics core at Michigan State University Research Technology Support Facility (Bio-Rad). Immunoblot analysis was preformed as above.

## Supporting Information

Figure S1 ***ADF4*** **expression does not change during the course of infection with** ***Pseudomonas syringae*** **expressing AvrPphB.** The expression levels of *ADF4* in Col-0, over time, when inoculated with *Pseudomonas syringae* expressing AvrPphB (*Pst* AvrPphB). Error bars represent mean ± SEM from two technical replicates of two independent biological replicates (n = 4). hpi = hours post inoculation. An unpaired student t-test with a 95% confidence interval was performed to determine if change over time was significant, where p>0.05 is considered not significant.
(TIF)

Figure S2 **Expression of 35S:RPS5-sYFP in** ***adf4*** **recovers the Hypersensitive Response.** (A) *RPS5* expression in two *adf4* mutant-complemented lines expressing 35S:RPS5-sYFP, *adf4*/35S:RPS5-sYFP-4 and *adf4*/35S:RPS5-sYFP-12. (B) Hypersensitive Response (HR) in *adf4*/35S:RPS5-sYFP-4 and *adf4*/35S:RPS5-sYFP-12 when challenged with *Pseudomonas syringae* expressing AvrPphB (*Pst* AvrPphB; left) and untreated (right).
(TIF)

Figure S3 **The** ***adf4*** **mutant does not have altered expression of other resistance genes.** The mRNA expression levels of *RPS2*, *RPM1*, *RPS4*, *RPS6* and *NDR1* in Col-0 and *adf4*. Error bars represent mean ± SEM from two technical replicates of two independent biological replicates (n = 4). hpi = hours post inoculation.
(TIF)

Figure S4 ***adf4*** **mutants are sensitive to fl22 in root length assay.** (A) Graphical representation of root lengths of Col-0 and *adf4* grown 10 days in the presence (+flg22) or absence (−flg22) of 10 nM flg22. Error bars represent mean ± SEM from two independent biological replicates (n = 32–46). Statistical significance was determined using two-way ANOVA, with Bonferroni post test, where ***p<0.001. (B) Col-0 and *adf4* seedlings grown for 10 days±10 nM flg22.
(TIF)

Figure S5 **Expression of** ***RPS5*** **mRNA is not affected by treatment with flg22, or by inoculation with the** ***hrpH*** **^−^ mutant of** ***Pseudomonas syringae*** **.** Real-time PCR analysis of *RPS5* mRNA accumulation in Col-0 and *adf4* following (A) flg22 treatment, mock inoculation or (B) dip-inoculation with the *hrpH* ^−^ mutant of *Pseudomonas syringae* (*Pst hrpH* ^−^). Expression was determined by qRT-PCR, utilizing amplification of *UBQ10* as an endogenous control. Error bars, representing mean ± SEM, were calculated from two technical replicates of two independent biological repeats (n = 4). Statistical significance was determined using two-way ANOVA as compared to Col-0, with Bonferroni post test, where ***p<0.001. hpi = hours post inoculation.
(TIF)

Figure S6 **Both Col-0 and** ***adf4*** **have induced** ***FRK1*** **expression when treated with elf18.** Relative expression levels of *FRK1* in Col-0 and *adf4* mutant plants, hand infiltrated with elf18. All expression values were determined by qRT-PCR, with amplification of *UBQ10* as an endogenous control. Error bars, representing mean ± SEM, are representative of two technical replicates of one biological repeat (n = 2). hpi = hours post inoculation.
(TIF)

Figure S7 **Increased** ***FRK1*** **expression in Col-0 and** ***adf4*** **when challenged by** ***Pst*** **AvrPphB-C98S, and HR phenotypes in Col-0,** ***adf4*** **, and** ***rps5-1*** **.** (A) The expression levels of *FRK1* in Col-0, *adf4* and *rps5-1* following dip-inoculation with *Pseudomonas syringae* expression the AvrPphB catalytic mutant C98S (*Pst* AvrPphB-C98S). All expression values were determined by qRT-PCR, with amplification of *UBQ10* as an endogenous control. Error bars, representing mean ± SEM, are representative of two technical replicates of three biological replicates (n = 6). hpi = hours post inoculation. (B) HR phenotypes in Col-0, *adf4* and *rps5-1* when hand inoculated with *Pst* AvrPphB-C98S.
(TIF)

Figure S8 **Estradiol-inducible expression of** ***avrPphB*** **in Col-0,** ***adf4*** **and** ***rps5-1*** **.** Induction of *avrPphB* expression in Col-0, *adf4* and *rps5-1* plants containing the estradiol-inducible *avrPphB* construct pER8:AvrPphB following 48 h pre-treatment with 100 µM estradiol. Expression values were determined by quantitative real-time PCR (qRT-PCR), with amplification of *UBQ10* as an endogenous control. Error bars, representing mean ± SEM, are representative two technical replicates of one biological repeat (n = 2).
(TIF)

Figure S9 ***RPS5*** **mRNA expression in additional** ***adf4*** **/35S:ADF4_S6A and** ***adf4*** **/35S:ADF4_S6D lines confirm observed** ***RPS5*** **expression is not due to positional effects of the transgene nor disproportionate levels of protein levels of protein expression.** (A) The expression level of *RPS5* in a second set of *adf4*/35S:ADF4_S6A (*adf4*/35S:ADF4_S6A-2) and *adf4*/35S:ADF4_S6D (*adf4*/35S:ADF4_S6D-2) transgenic lines, as compared to the first line shown in Figure 4A. All expression values were determined by quantitative real-time PCR (qRT-PCR), with amplification of *UBQ10* as an endogenous control. Error bars, representing mean ± SEM, are representative of two technical replicates of one biological repeat (n = 2). hpi = hours post inoculation. (B) Relative protein levels of ADF4_S6A and ADF4_S6D in *adf4*/35S:ADF4_S6A and *adf4*/35S:ADF4_S6D as determined by western blot when probed with anti-T7-HRP. Ponceau blot is shown to demonstrate equal loading.
(TIF)

Figure S10 ***FRK1*** **expression in** ***adf4*** **/35S:ADF4_S6A and** ***adf4*** **/35S:ADF4_S6D lines confirm link between** ***RPS5*** **expression and** ***FRK1*** **in the presence of** ***Pseudomonas syringae*** **expressing AvrPphB.** Relative expression levels of *FRK1* mRNA following dip-inoculation with *Pseudomonas syringae* expressing AvrPphB (*Pst* AvrPphB) in *adf4*/35S:ADF4_S6A and *adf4*/35S:ADF4_S6D determined by quantitative real-time PCR (qRT-PCR), with amplification of *UBQ10* as an endogenous control. Error bars, representing mean ± SEM, are representative of two technical replicates of two independent biological replicates (n = 4). Statistical significance was determined using two-way ANOVA as compared to Col-0, with Bonferroni post test, where *p<0.05. hpi = hours post inoculation.
(TIF)

Table S1 **qRT-PCR primers used in this study.**
(DOCX)

Table S2 **Mathematical equations used for co-localization overlap coefficient determination.**
(DOCX)
