# Supplementary figures and images for: Arabidopsis Actin-Depolymerizing Factor-4 Links Pathogen Perception, Defense Activation and Transcription to Cytoskeletal Dynamics
Source: PLoS Pathog. 2012 Nov 8;8(11):e1003006. doi: 10.1371/journal.ppat.1003006 (PMC3493479; doi:10.1371/journal.ppat.1003006)

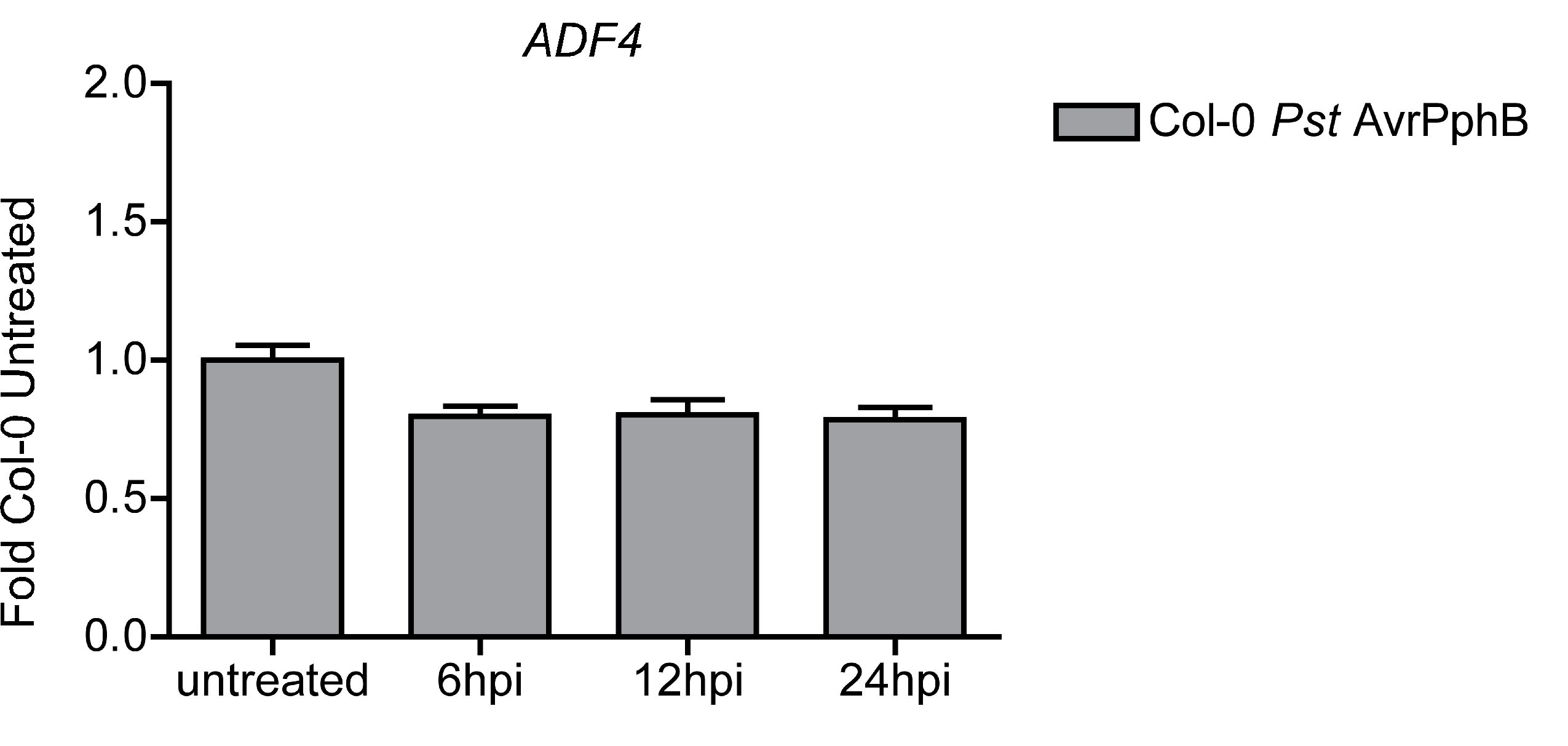

Supplement: Figure S1 — ADF4 expression does not change during the course of infection with Pseudomonas syringae expressing AvrPphB. The expression levels of ADF4 in Col-0, over time, when inoculated with Pseudomonas syringae expressing AvrPphB (Pst AvrPphB). Error bars represent mean ± SEM from two technical replicates of two independent biological replicates (n = 4). hpi = hours post inoculation. An unpaired student t-test with a 95% confidence interval was performed to determine if change over time was significant, where p>0.05 is considered not significant. (TIF) [file ppat.1003006.s001.tif]

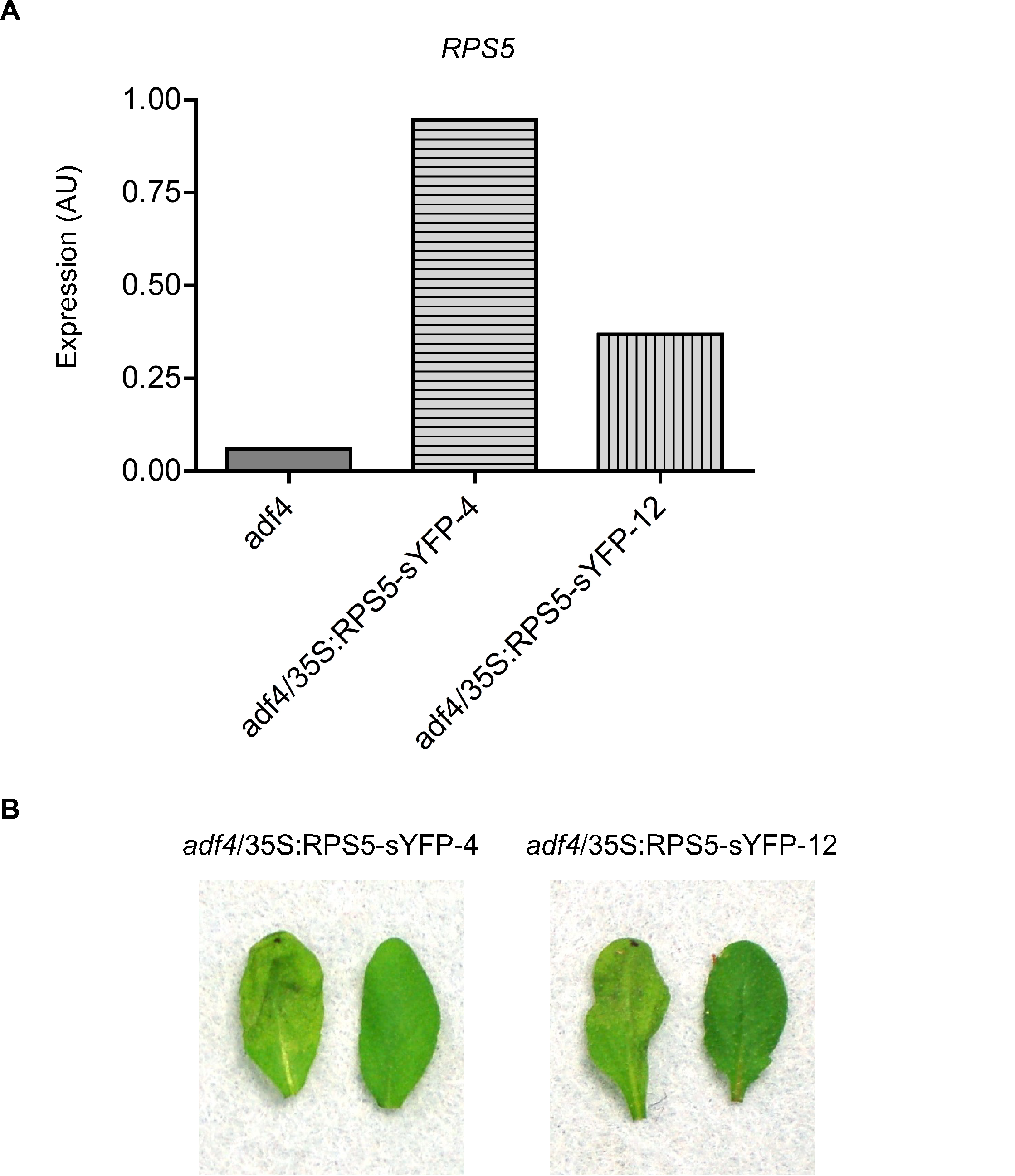

Supplement: Figure S2 — Expression of 35S:RPS5-sYFP in adf4 recovers the Hypersensitive Response. (A) RPS5 expression in two adf4 mutant-complemented lines expressing 35S:RPS5-sYFP, adf4/35S:RPS5-sYFP-4 and adf4/35S:RPS5-sYFP-12. (B) Hypersensitive Response (HR) in adf4/35S:RPS5-sYFP-4 and adf4/35S:RPS5-sYFP-12 when challenged with Pseudomonas syringae expressing AvrPphB (Pst AvrPphB; left) and untreated (right). (TIF) [file ppat.1003006.s002.tif]

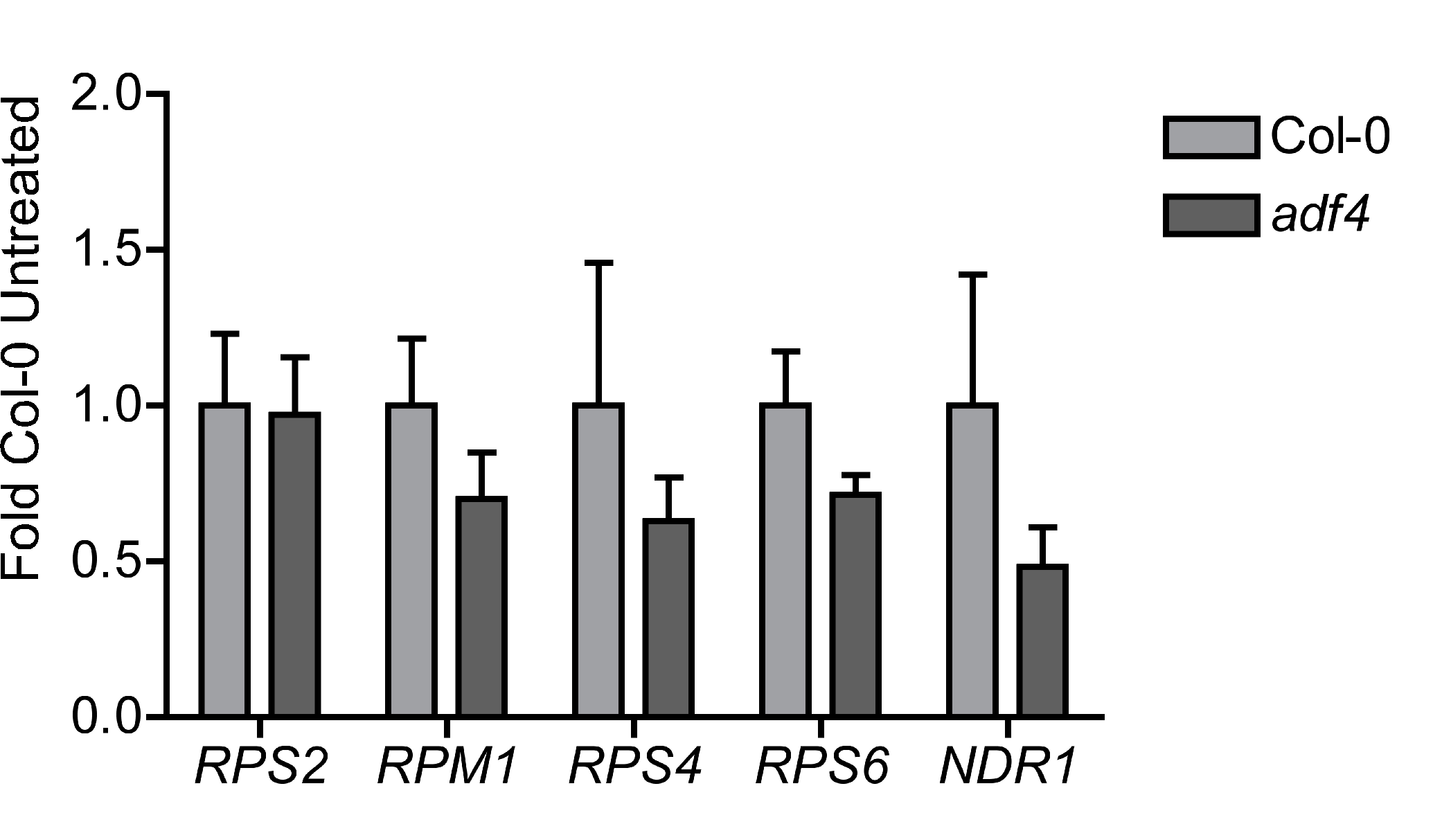

Supplement: Figure S3 — The adf4 mutant does not have altered expression of other resistance genes. The mRNA expression levels of RPS2, RPM1, RPS4, RPS6 and NDR1 in Col-0 and adf4. Error bars represent mean ± SEM from two technical replicates of two independent biological replicates (n = 4). hpi = hours post inoculation. (TIF) [file ppat.1003006.s003.tif]

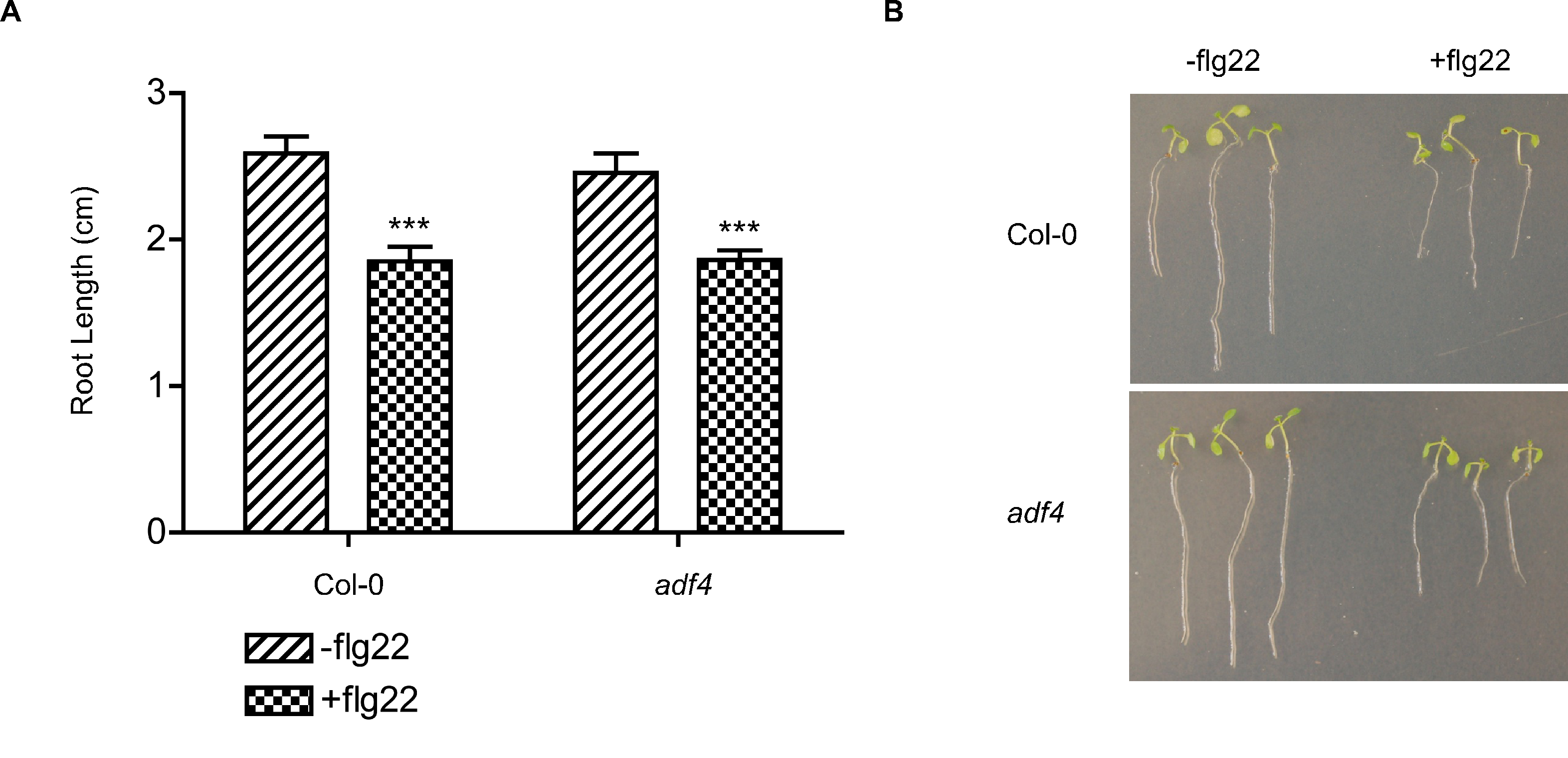

Supplement: Figure S4 — adf4 mutants are sensitive to fl22 in root length assay. (A) Graphical representation of root lengths of Col-0 and adf4 grown 10 days in the presence (+flg22) or absence (−flg22) of 10 nM flg22. Error bars represent mean ± SEM from two independent biological replicates (n = 32–46). Statistical significance was determined using two-way ANOVA, with Bonferroni post test, where ***p<0.001. (B) Col-0 and adf4 seedlings grown for 10 days±10 nM flg22. (TIF) [file ppat.1003006.s004.tif]

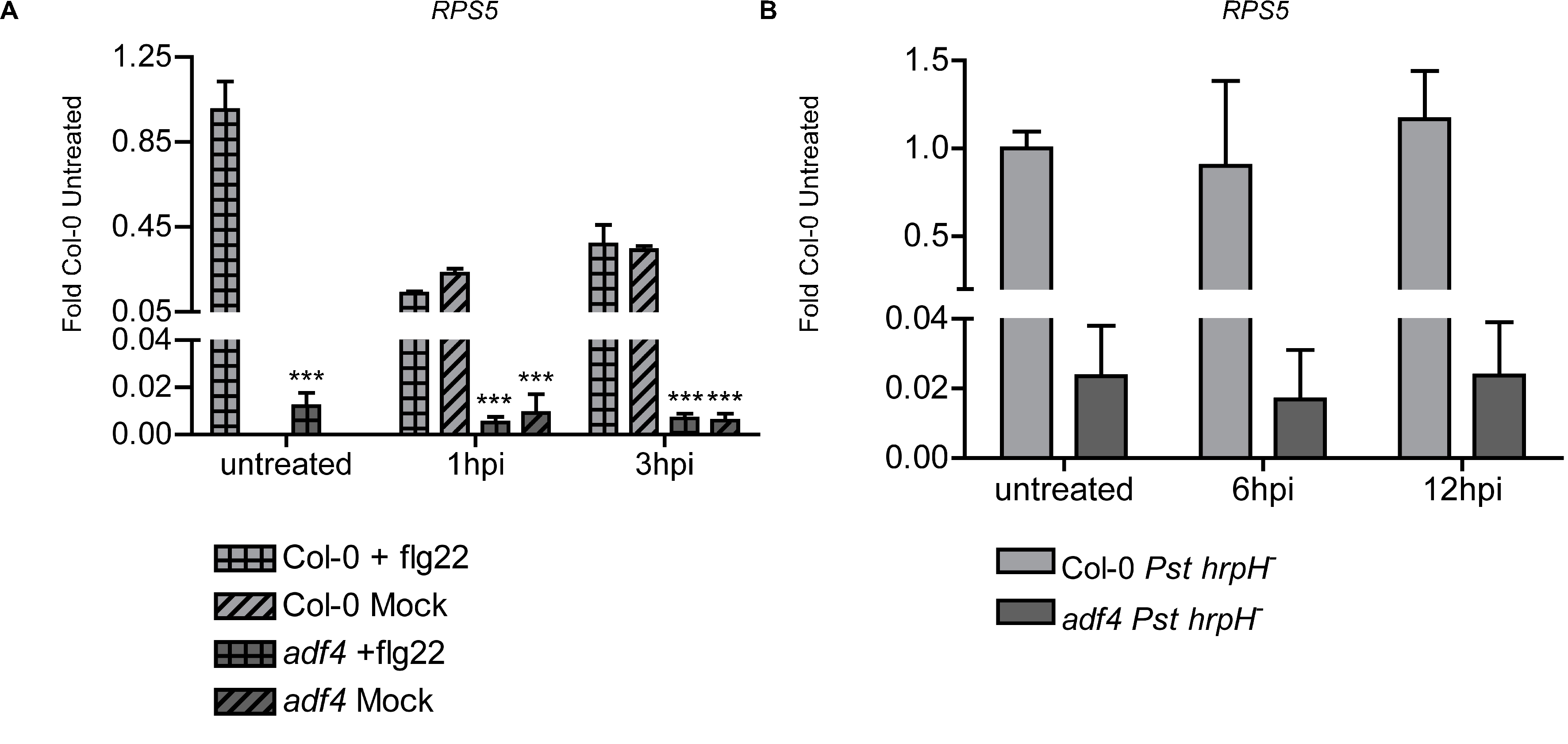

Supplement: Figure S5 — Expression of RPS5 mRNA is not affected by treatment with flg22, or by inoculation with the hrpH − mutant of Pseudomonas syringae . Real-time PCR analysis of RPS5 mRNA accumulation in Col-0 and adf4 following (A) flg22 treatment, mock inoculation or (B) dip-inoculation with the hrpH − mutant of Pseudomonas syringae (Pst hrpH −). Expression was determined by qRT-PCR, utilizing amplification of UBQ10 as an endogenous control. Error bars, representing mean ± SEM, were calculated from two technical replicates of two independent biological repeats (n = 4). Statistical significance was determined using two-way ANOVA as compared to Col-0, with Bonferroni post test, where ***p<0.001. hpi = hours post inoculation. (TIF) [file ppat.1003006.s005.tif]

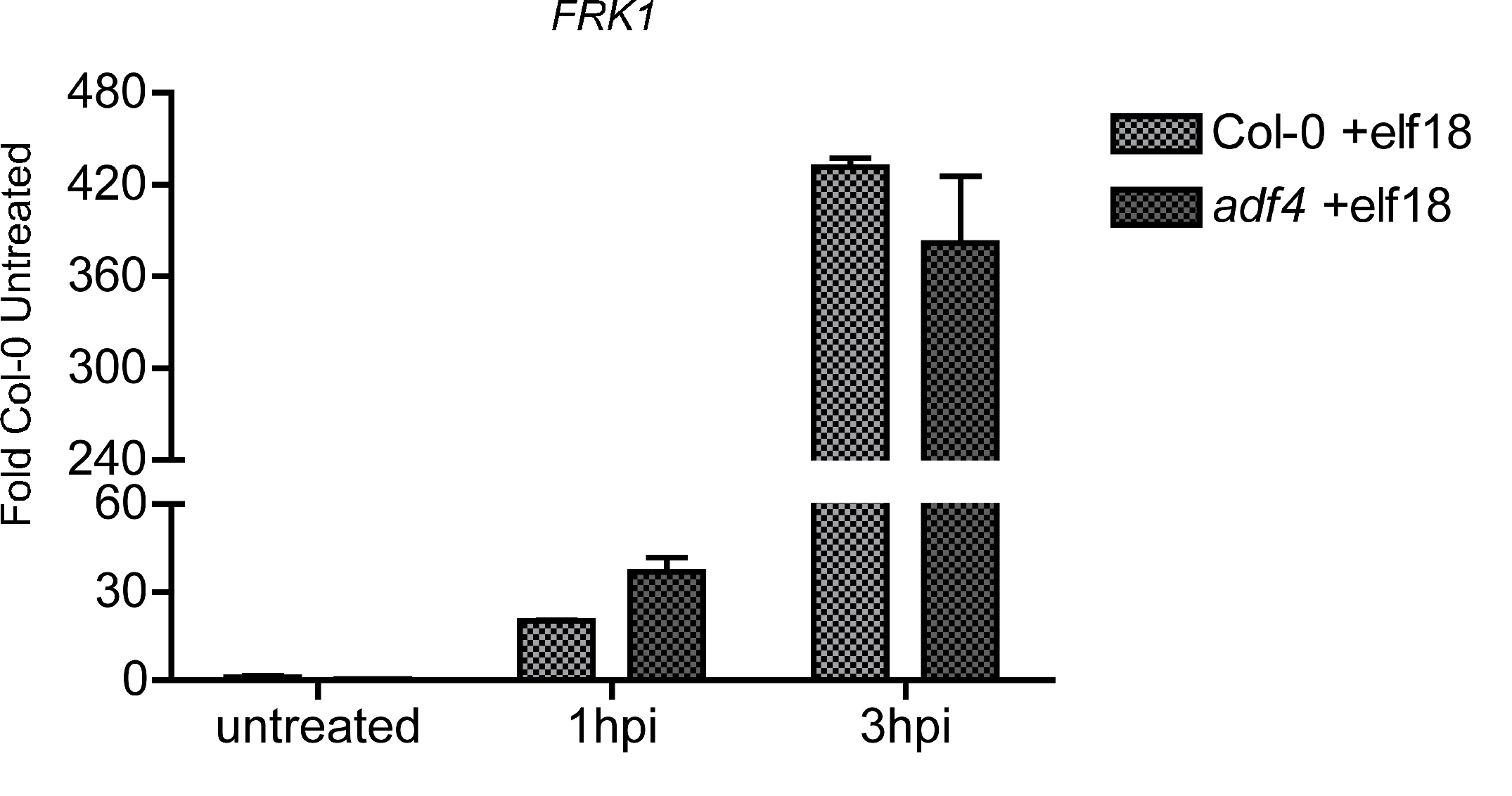

Supplement: Figure S6 — Both Col-0 and adf4 have induced FRK1 expression when treated with elf18. Relative expression levels of FRK1 in Col-0 and adf4 mutant plants, hand infiltrated with elf18. All expression values were determined by qRT-PCR, with amplification of UBQ10 as an endogenous control. Error bars, representing mean ± SEM, are representative of two technical replicates of one biological repeat (n = 2). hpi = hours post inoculation. (TIF) [file ppat.1003006.s006.tif]

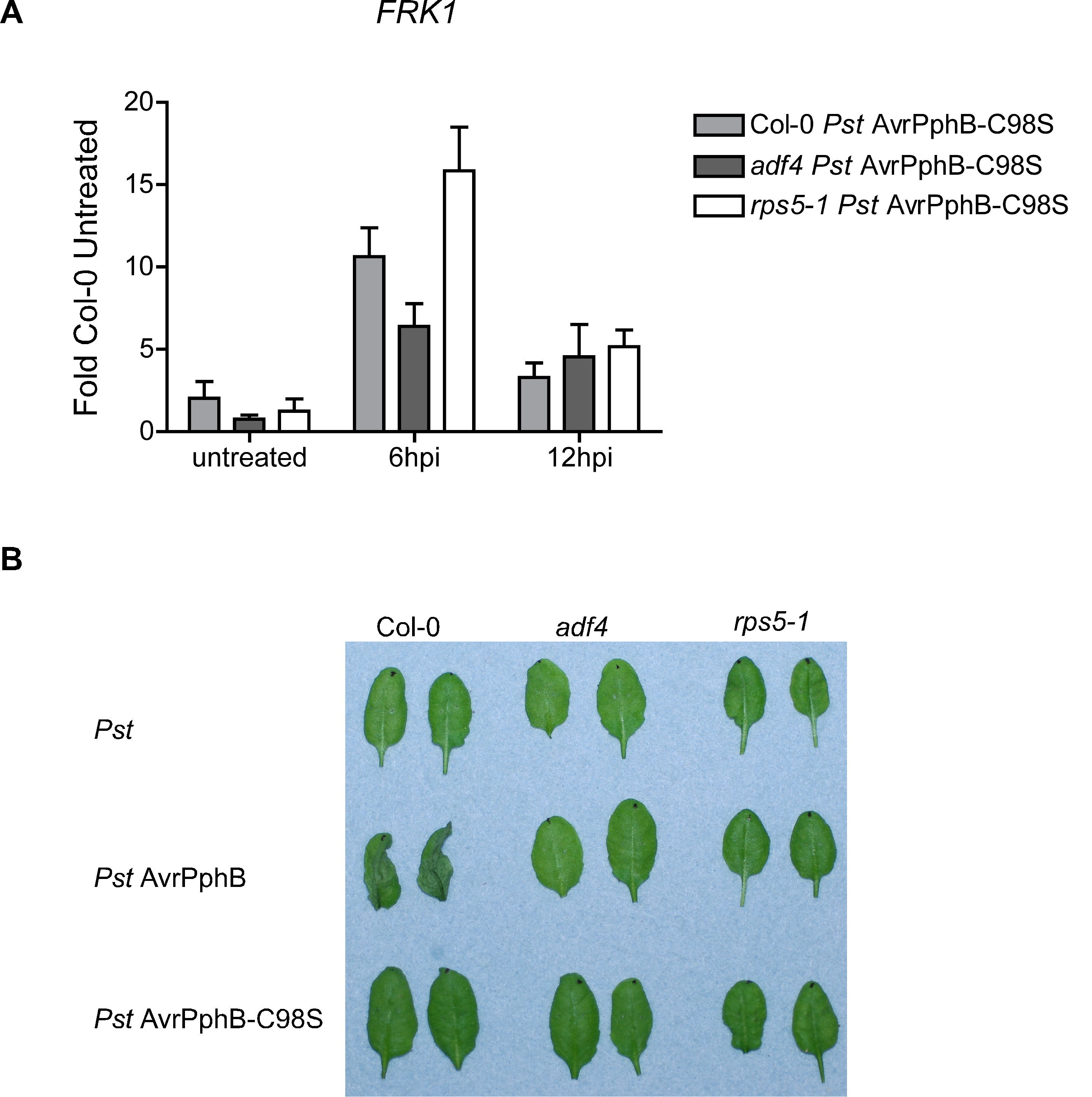

Supplement: Figure S7 — Increased FRK1 expression in Col-0 and adf4 when challenged by Pst AvrPphB-C98S, and HR phenotypes in Col-0, adf4 , and rps5-1 . (A) The expression levels of FRK1 in Col-0, adf4 and rps5-1 following dip-inoculation with Pseudomonas syringae expression the AvrPphB catalytic mutant C98S (Pst AvrPphB-C98S). All expression values were determined by qRT-PCR, with amplification of UBQ10 as an endogenous control. Error bars, representing mean ± SEM, are representative of two technical replicates of three biological replicates (n = 6). hpi = hours post inoculation. (B) HR phenotypes in Col-0, adf4 and rps5-1 when hand inoculated with Pst AvrPphB-C98S. (TIF) [file ppat.1003006.s007.tif]

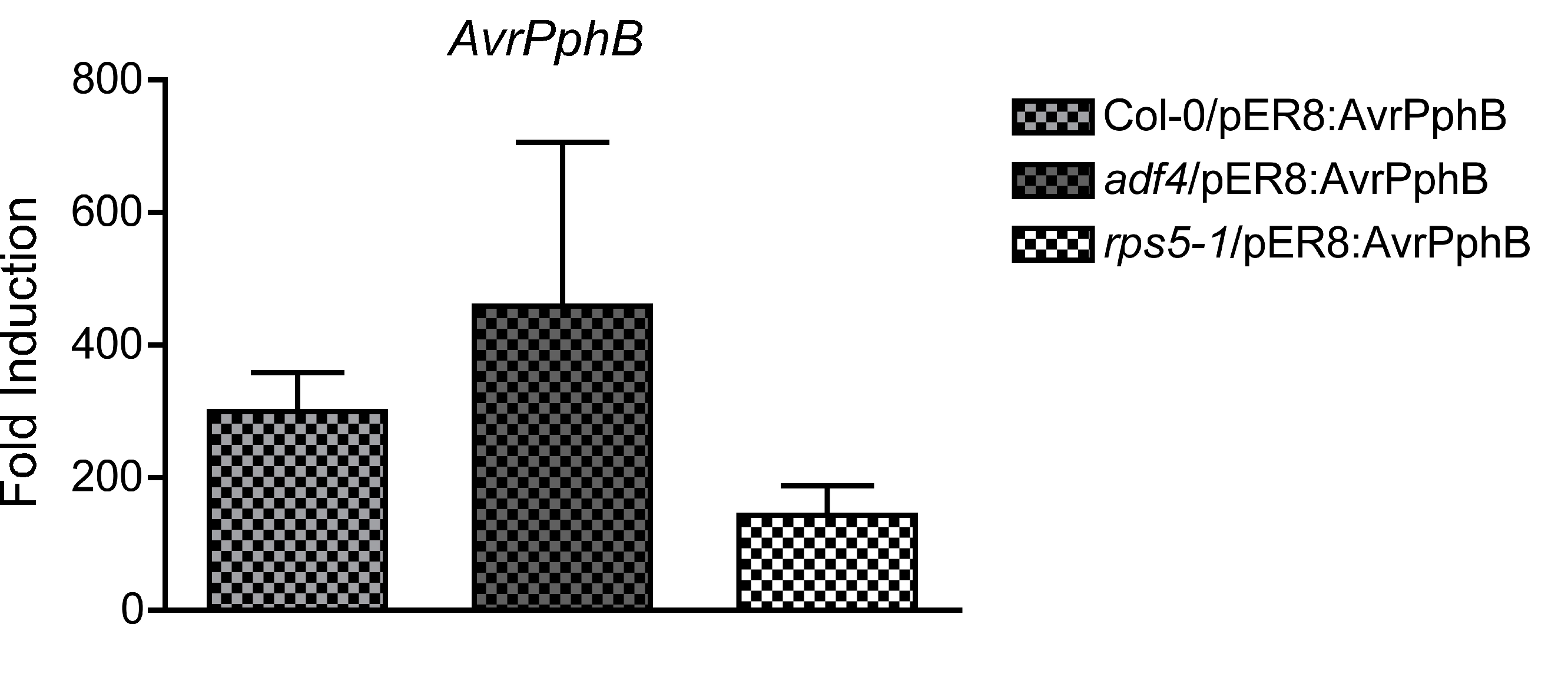

Supplement: Figure S8 — Estradiol-inducible expression of avrPphB in Col-0, adf4 and rps5-1 . Induction of avrPphB expression in Col-0, adf4 and rps5-1 plants containing the estradiol-inducible avrPphB construct pER8:AvrPphB following 48 h pre-treatment with 100 µM estradiol. Expression values were determined by quantitative real-time PCR (qRT-PCR), with amplification of UBQ10 as an endogenous control. Error bars, representing mean ± SEM, are representative two technical replicates of one biological repeat (n = 2). (TIF) [file ppat.1003006.s008.tif]

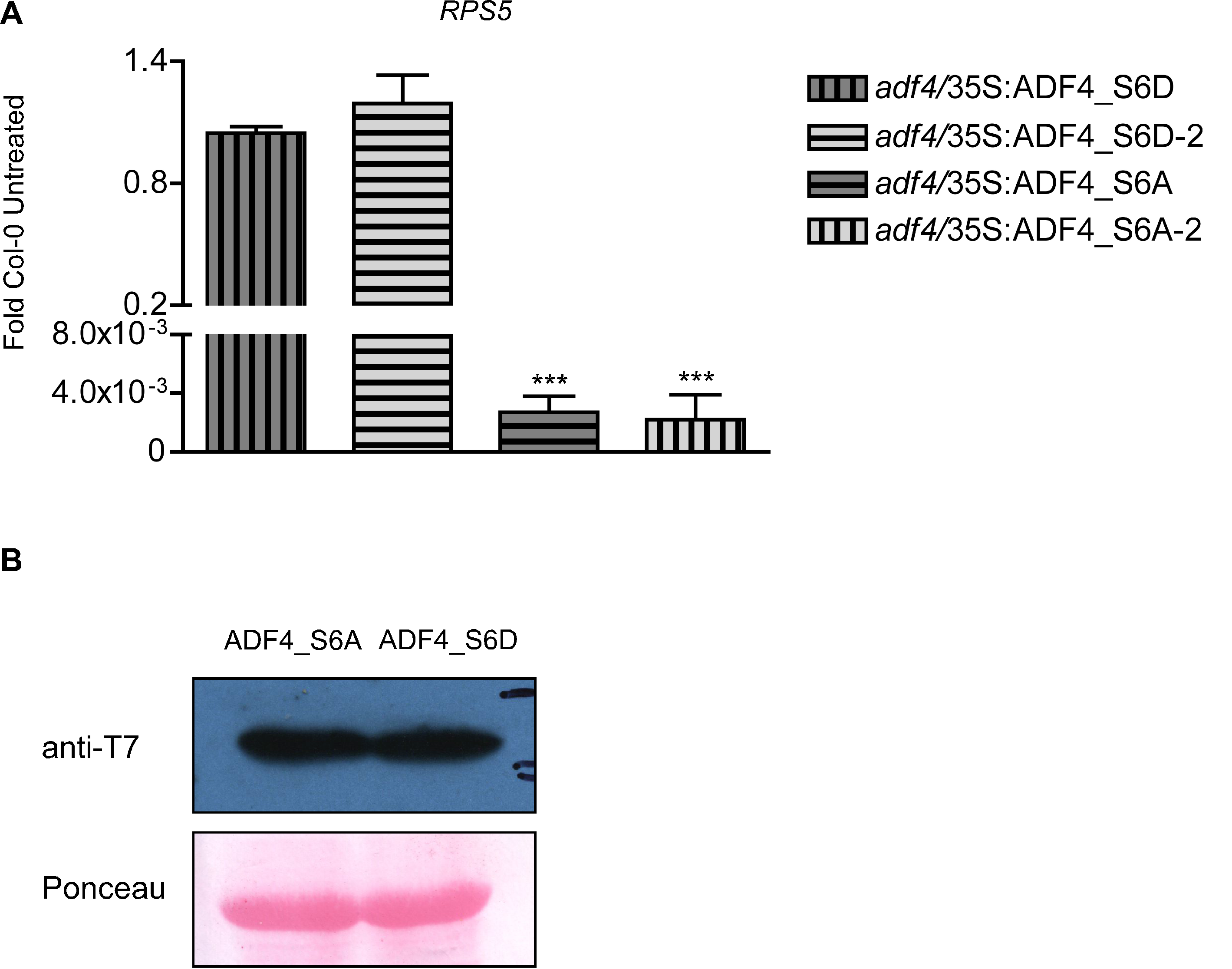

Supplement: Figure S9 — RPS5 mRNA expression in additional adf4 /35S:ADF4_S6A and adf4 /35S:ADF4_S6D lines confirm observed RPS5 expression is not due to positional effects of the transgene nor disproportionate levels of protein levels of protein expression. (A) The expression level of RPS5 in a second set of adf4/35S:ADF4_S6A (adf4/35S:ADF4_S6A-2) and adf4/35S:ADF4_S6D (adf4/35S:ADF4_S6D-2) transgenic lines, as compared to the first line shown in Figure 4A. All expression values were determined by quantitative real-time PCR (qRT-PCR), with amplification of UBQ10 as an endogenous control. Error bars, representing mean ± SEM, are representative of two technical replicates of one biological repeat (n = 2). hpi = hours post inoculation. (B) Relative protein levels of ADF4_S6A and ADF4_S6D in adf4/35S:ADF4_S6A and adf4/35S:ADF4_S6D as determined by western blot when probed with anti-T7-HRP. Ponceau blot is shown to demonstrate equal loading. (TIF) [file ppat.1003006.s009.tif]

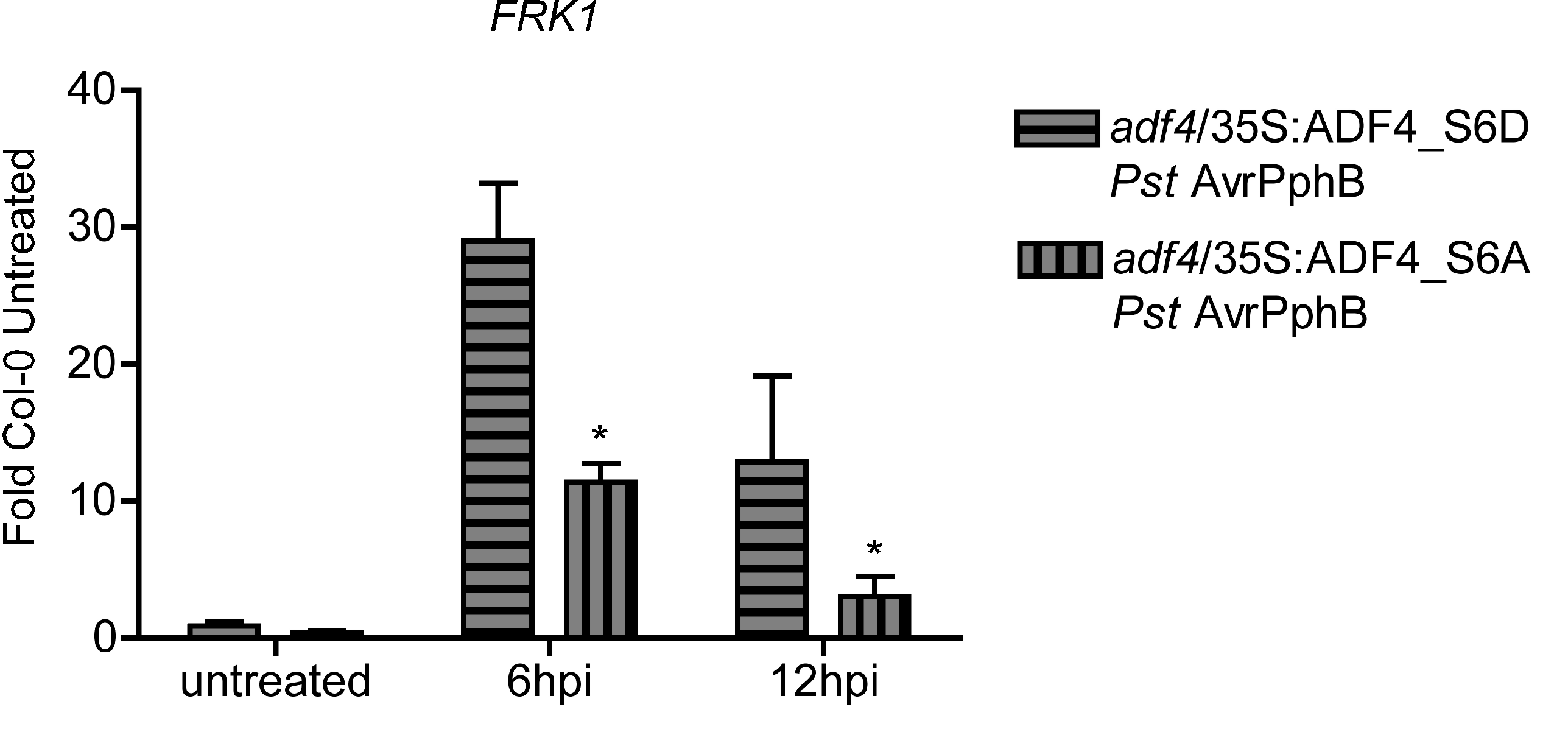

Supplement: Figure S10 — FRK1 expression in adf4 /35S:ADF4_S6A and adf4 /35S:ADF4_S6D lines confirm link between RPS5 expression and FRK1 in the presence of Pseudomonas syringae expressing AvrPphB. Relative expression levels of FRK1 mRNA following dip-inoculation with Pseudomonas syringae expressing AvrPphB (Pst AvrPphB) in adf4/35S:ADF4_S6A and adf4/35S:ADF4_S6D determined by quantitative real-time PCR (qRT-PCR), with amplification of UBQ10 as an endogenous control. Error bars, representing mean ± SEM, are representative of two technical replicates of two independent biological replicates (n = 4). Statistical significance was determined using two-way ANOVA as compared to Col-0, with Bonferroni post test, where *p<0.05. hpi = hours post inoculation. (TIF) [file ppat.1003006.s010.tif]
