# Supplementary material for: Arabidopsis Actin-Depolymerizing Factor-4 Links Pathogen Perception, Defense Activation and Transcription to Cytoskeletal Dynamics
Source: PLoS Pathog. 2012 Nov 8;8(11):e1003006. doi: 10.1371/journal.ppat.1003006 (PMC3493479; doi:10.1371/journal.ppat.1003006)
Supplement: Table S1 — qRT-PCR primers used in this study. (DOCX) [file ppat.1003006.s011.docx]

**Table S1**

| **Description** | **Equation** |
| --- | --- |
| *Overlap coefficient according to Manders (R)* | $R=\frac{\sum_{i} S1_{i}\cdot S2_{i}}{\sqrt{\sum_{i} (S1_{i})^{2}\cdot\sum_{i} (S2_{i})^{2}}}$ |
| *Co-localization coefficient m_1_* | **$m_{1}=\frac{\sum_{i} S1_{i,coloc}}{\sum_{i} S1_{i}}$** |
| *Co-localization coefficient m_2_* | **$m_{2}=\frac{\sum_{i} S2_{i,coloc}}{\sum_{i} S2_{i}}$** |
