# Supplementary material for: Arabidopsis Actin-Depolymerizing Factor-4 Links Pathogen Perception, Defense Activation and Transcription to Cytoskeletal Dynamics
Source: PLoS Pathog. 2012 Nov 8;8(11):e1003006. doi: 10.1371/journal.ppat.1003006 (PMC3493479; doi:10.1371/journal.ppat.1003006)
Supplement: Table S2 — Mathematical equations used for co-localization overlap coefficient determination. (DOCX) [file ppat.1003006.s012.docx]

**Table S2**

| Locus Tag | Gene | Forward Primer (5´-3´) | Reverse Primer (5´-3´) |
| --- | --- | --- | --- |
| AT1G12220 | *RPS5* | GTTGTCATGGTCTAAAGACATTTG | GTACAAATCCAATGATCACTAACCA |
| AT5G13160 | *PBS1* | TCAATGTGCATCCAAGAACAGGCG | AATTTACTTCCCGAGCCACCTCCA |
| AT4G26090 | *RPS2* | GGCGGAGAGAAGAGGACATA | CAGCTTCGTCCCTCTAGACC |
| AT3G07040 | *RPM1* | TCGCGGAGAAGGGAGTGTGGA | GAAGCTTGCCTTGGCCGCCT |
| AT4G05320 | *UBQ10* | AGAAGTTCAATGTTTCGTTTCATGTAA | TTACGAATCCGAGGGAGCCATTG |
| AT2G19190 | *FRK1* | CGGTCAGATTTCAACAGTTGTC | AATAGCAGGTTGGCCTGTAATC |
| AT5G45250 | *RPS4* | CCTAACATTATGGGCATCATCA | CCGCCTTCACAATTTCATTGA |
| AT5G46470 | *RPS6* | GGTCAATTCAACTACGATCACG | GTTATCCAGGGATGGGACAT |
| AT3G20600 | *NDR1* | CGGTTTTACGAGCGGTTTTG | CCAACTTCAACCCCATACCTC |
| AT3G45640 | *MPK3* | TGACGTTTGACCCCAACAGA | CTGTTCCTCATCCAGAGGCTG |
| AT2G43790 | *MPK6* | CCGACAGTGCATCCTTTAGCT | TGGGCCAATGCGTCTAAAAC |
| n/a | *AvrPphB* | GGTGGCAGTGCGCAATTGGG | TCCCTCACAGGAGCACGCGAT |
